# Understanding Isotope Substitution Effects in Water Using the Potential Energy Landscape Formalism for Quantum Liquids

**DOI:** 10.1021/acs.jctc.5c01325

**Published:** 2025-11-18

**Authors:** Ali Eltareb, Yang Zhou, Gustavo E. Lopez, Nicolas Giovambattista

**Affiliations:** † Department of Physics, 2037 Brooklyn College of the City University of New York, Brooklyn, New York 11210, United States; ‡ Ph.D. Program in Physics, 14772 The Graduate Center of the City University of New York, New York, New York 10016, United States; § Department of Chemistry, Lehman College of the City University of New York, Bronx, New York 10468, United States; ∥ Ph.D. Program in Chemistry, The Graduate Center of the City University of New York, New York, New York 10016, United States

## Abstract

Isotope substitution effects are known to alter the thermodynamic, dynamic, and structural properties of water, particularly, at low temperatures. In this work, we perform path-integral (PI) computer simulations of H_2_O, HDO, D_2_O, and T_2_O, and provide a rigorous description of the *H* ↔ *D* ↔ *T* isotope substitution effects in water based on the potential energy landscape (PEL) formalism for quantum liquids. Our PI computer simulations at 200 ≤ *T* ≤ 400 K and *v* = 18.0 cm^3^/mol (corresponding to a density for H_2_O of ρ = 1.00 g/cm^3^) indicate that the same potential energy minima (inherent structures, IS) are present in the PEL of *quantum* water and its isotopes (sampled in PI computer simulations). These IS are also isomorphic to (and can be obtained from) the IS of *classical* water (sampled in classical computer simulations). Isotope substitutions in water (*H* ↔ *D* ↔ *T*) have the only effect of altering the curvature (and shape) of water’s PEL basins about the corresponding IS. Specifically, the PEL basins become wider along the sequence H_2_O → HDO → D_2_O → T_2_O, as the atoms delocalization become less pronounced. From a thermodynamic point of view, we find that water and its isotopes *at a given temperature*, sample different IS of the corresponding PEL, with different curvatures, explaining the subtle variations in the properties of H_2_O, HDO, D_2_O, and T_2_O. It is also shown that the Adam–Gibbs relation remains valid for water and its isotopes implying that, in all these cases, the topography of the PEL controls the dynamics of the system (molecular diffusion) at a given temperature. Overall, this work shows that the PEL formalism for quantum liquids provides an intuitive and rigorous theoretical framework within statistical mechanics that can be used to describe isotopic substitution effects in liquids.

## Graphical abstract

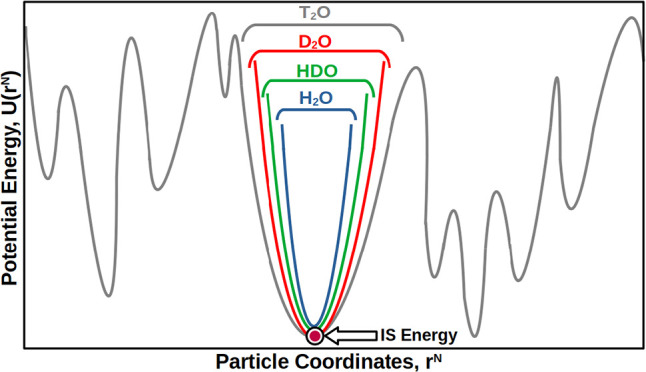

## 1 Introduction

Water and its isotopes (more precisely, water isotopologues) play a fundamental role across the physical, chemical, and biological sciences.^1–6^ For example, the differences in the properties of H_2_O and D_2_O are crucial in NMR and spectroscopy techniques, commonly used to characterize the behavior of aqueous solutions and biological samples.^7,8^ Hence, it is not surprising that the unique structural, dynamic, and thermodynamic properties of water, and their sensitivity to isotopic substitution, have been the subject of extensive investigation. Unlike most liquids, water exhibits a number of anomalous behaviors, such as a density maximum, and increases in isothermal compressibility and heat capacity upon isobaric cooling.^9–13^ These anomalies also persist across the isotopes of water (H_2_O, D_2_O, and T_2_O), though they occur at different temperatures.^2,14–16^ For example, D_2_O has a temperature of maximum density and melting point that is approximately 8 and 4 K higher than those of H_2_O, respectively.^2,17^

The isotope substitution effects (ISE) in the thermodynamic properties of water can be rationalized in terms of the corresponding hydrogen-bond (HB) strength which, ultimately, arise from nuclear quantum effects (NQE) (atoms delocalization).^18–20^ For example, the slightly higher melting temperature of D_2_O relative to H_2_O indicates that the hydrogen-bonds are slightly stronger in D_2_O than in H_2_O. Indeed, computer simulations support this view, even when the differences in the HB strength among the water isotopes are small.^17,18,21–24^ Consistent with this view, experiments also show that proteins exhibit enhanced rigidity and folding stability when D_2_O is used as the solvent instead of H_2_O.^7,25,26^ Phenomenological models have been proposed to rationalize the ISE in water. These include simple temperature scaling^27–29^ and, more recently, a corresponding states analysis based on the existence of a liquid–liquid critical point in supercooled water.^30,31^ The corresponding states approach is particularly attractive because it is rooted on the idea that water and its isotopes exhibit common behavior when compared at properly scaled conditions. While such macroscopic approaches can effectively capture some of the trends between H_2_O and D_2_O, they do not provide a microscopic or statistical mechanical understanding.

In this work, we combine path-integral computer simulations and the potential energy landscape (PEL) formalism for quantum liquids to describe the role of H ↔ D ↔ T isotopes substitution on the properties of water. By relating the ISE with the topography of the PEL of water and its isotopes, this approach provides a statistical mechanics/thermodynamics understanding of the ISE in water. The PEL formalism is an approach within statistical mechanics that was originally introduced to study *classical* liquids and glasses.^32–34^ Briefly, for a *classical* system composed of *N* atoms, the PEL is the surface in (3*N* + 1)-dimensional space defined by the potential energy function of the system as a function of the atoms coordinates (see Section 2). Since in its original form the PEL formalism is based on classical statistical mechanics, the mass of the atoms do not play any role and hence, NQE are ignored, rendering the (classical) formalism limited and insufficient to study isotopic substitution effects. By using the path-integral formulation of statistical mechanics, the PEL formalism has been recently extended to study quantum liquids.^35–38^ In this approach, the original (quantum) liquid is mapped onto a classical system of ring-polymers, each composed of *n*_*b*_ beads (Section 2). Accordingly, the PEL associated with the quantum liquid is identified with the (classical) PEL of the corresponding ring-polymer system (RP-PEL), and it is defined by the potential energy function of the ring-polymer system as a function of the 3*Nn*_*b*_ beads coordinates. Contrary to the classical case, the PEL of the quantum liquid/ring-polymer system depends on the temperature and, particularly, on the atoms mass, providing a natural foundation for studying the NQE arising from the atoms delocalization including isotopic substitution.^37,39^ Our study is based on path-integral computer simulations of H_2_O, HDO, D_2_O, and T_2_O using the q-TIP4P/F model over a wide temperature range, at constant volume *v* = 18.0 cm^3^/mol, which corresponds to a density of ρ = 1.0 g/cm^3^ for H_2_O. We note that the thermodynamic, structural, and dynamical properties of these isotopes as a function of temperature are very similar along the *P* = 0.1 MPa, and *v* = 18.0 cm^3^/mol isochore.^18^

The structure of this work is as follows. In Section 2, we present a brief review of the PEL formalism for quantum liquids, adapted to the case of q-TIP4P/F water. We also introduce the Gaussian and harmonic approximations of the PEL, and describe how corrections to the PEL formalism can be introduced due to the PEL basins anharmoncities. The computational details are given in Section 3 and the results are presented in Section 4. Section 5 includes a summary and discussion.

## 2 Potential Energy Landscape Formalism For A Quantum Flexible Water Model

In this section, we provide a brief review of the PEL formalism for a flexible water model, such as the q-TIP4P/F model, where the intermolecular and intramolecular forces are determined solely by the positions of the oxygen and hydrogen atoms. The PEL formalism presented here holds for liquids that obey quantum mechanics and is based on the path-integral formulation of statistical mechanics; see refs 35–38 for additional details.

*The Canonical Partition Function*: The canonical partition function for a quantum system composed of *N* water molecules at constant volume *V* and temperature *T* is given by^40,41^

1 Q(N,V,T)=Tr(ρ̂)

where Tr­(ρ̂) is the trace of the density operator ρ̂ = exp­(−*βĤ*) and *Ĥ* is the Hamiltonian operator of the system,

2 $$Ĥ=\sum_{i=1}^{n}\frac{{\mathrm{p̂}}_{i}^{2}}{2m_{i}}+U\left({\mathrm{r̂}}_{1},{\mathrm{r̂}}_{2},..,{\mathrm{r̂}}_{n}\right)$$

­(***r̂***_*i*_, ***p̂***_*i*_) are the position and momentum operators associated with atom *i* = 1, 2, .., *n*, where *n* = 3*N* is the total number of atoms (O and H) in the system; *m*_*i*_ is the corresponding mass, and $\beta =\frac{1}{k_{B}T}$ where *k*_*B*_ is the Boltzmann’s constant. Using the path-integral formulation of statistical mechanics,^40,41^ it can be shown that eq 1 is mathematically identical to the canonical partition function of a *classical* system of *n* distinguishable ring-polymers composed of *n*_*b*_ distinguishable beads. Specifically, eq 1 can be written as follows

3 $$Q\left(N,V,T\right)=\lim_{n_{b}\;\to \infty }\frac{1}{h^{3n_{b}n}}\int_{V}\left[\prod_{i=1}^{n}dr_{i}^{1}...dr_{i}^{n_{b}}\right]\int_{-\infty }^{\infty }\left[\prod_{i=1}^{n}dp_{i}^{1}...dp_{i}^{n_{b}}\right]\exp\left[-\beta H_{\mathrm{RP}}\left(R,P\right)\right]$$

where the Hamiltonian of the *classical* ring-polymer system, $H_{\mathrm{RP}}\left(R,P\right)$ , is given by

4 $$H_{\mathrm{RP}}\left(R,P\right)=\sum_{i=1}^{n}\sum_{k=1}^{n_{b}}\frac{{\left(p_{i}^{k}\right)}^{2}}{2m_{i}^{\prime }}+\sum_{i=1}^{n}\sum_{k=1}^{n_{b}}\frac{1}{2}k_{i}^{\mathrm{sp}}{\left(r_{i}^{k+1}-r_{i}^{k}\right)}^{2}+\frac{1}{n_{b}}\sum_{k=1}^{n_{b}}U\left(r_{1}^{k},r_{2}^{k},...,r_{n}^{k}\right)$$

In this expression, (***r***_*i*_^*k*^, ***p***_*i*_^*k*^) are the vector position and momentum of bead *k* = 1,2, ..., *n*_*b*_ belonging to ring-polymer *i* = 1, 2, ..., *n*, and ***r***_*i*_^*n*_*b*_+1^ = ***r***_*i*_^1^ for all values of *i* (since the system is composed of *ring*-polymers); for simplicity, we denote ***R*** = (***r***_1_^1^, ***r***_1_^2^, ..., ***r***_1_^*n*_*b*_^; ...; ***r***_*n*_^1^, ***r***_*n*_^2^, ..., ***r***_*n*_^*n*_*b*_^) and ***P*** = (***p***_1_^1^, ***p***_1_^2^, ..., ***p***_1_^*n*_*b*_^;...; ***p***_*n*_^1^, ***p***_*n*_^2^, ..., ***p***_*n*_^*n*_*b*_^). In eq 4, $k_{i}^{\mathrm{sp}}=\frac{m_{i}n_{b}}{{\left(\hbar \beta \right)}^{2}}$ is the (temperature-dependent) spring constant of ring-polymer *i* associated with atom *i* (O or H) of the system; *m*_*i*_^′^ = *n*_*b*_ *m*_*i*_ is the mass of the beads belonging to ring-polymer/atom *i* (with mass *m*_*i*_). We note that *k*_*i*_^*sp*^ ∝ *m*_*i*_ and hence, the partition function as well as the thermodynamic properties of water are expected to vary upon isotope substitutions. It follows from eq 4, that the potential energy of the ring-polymer system is given by

5 $$U_{\mathrm{RP}}\left(R\right)=\sum_{i=1}^{n}\sum_{k=1}^{n_{b}}\frac{1}{2}k_{i}^{\mathrm{sp}}{\left(r_{i}^{k+1}-r_{i}^{k}\right)}^{2}+\frac{1}{n_{b}}\sum_{k=1}^{n_{b}}U\left(r_{1}^{k},r_{2}^{k},...,r_{n}^{k}\right)$$

Accordingly, as explained in refs 36,37 , eq 5 defines a PEL for water in the presence of NQE (for a fix value of *n*_*b*_).

*Simplifying the Canonical Partition Function*: The main idea of the PEL formalism is to partition the PEL (defined by eq 5) into basins,^32–34^ and to rewrite the canonical partition function in eq 3 as the sum of the canonical partition function of the individual basins. To each local minimum of the PEL (or, inherent structure, IS) there is one and only one basin; the corresponding basin is defined as the set of points of the PEL that lead to the given IS by steepest descent (i.e., by potential energy minimization).^32,42–44^ As explained in refs 36,37 , eq 3 can then be rewritten (exactly) as

6 $$Q\left(N,V,T\right)=\sum_{e_{\mathrm{IS}}}e^{-\beta \left[e_{\mathrm{IS}}-TS_{\mathrm{IS}}\left(N,V,T,e_{\mathrm{IS}}\right)+F_{\mathrm{vib}}\left(N,V,T,e_{\mathrm{IS}}\right)\right]}$$

where the sum runs over all the IS of the PEL with energy *e*_*IS*_. In eq 6, *S*_*IS*_(*N*, *V*, *T*, *e*_*IS*_) is the configurational entropy of the system and quantifies the number of IS in the PEL with energy *e*_*IS*_ [at constant (*N*, *V*, *T*) ]. Specifically

7 $$S_{\mathrm{IS}}\left(N,V,T,e_{\mathrm{IS}}\right)\equiv k_{B}\ln\left[\Omega_{\mathrm{IS}}\left(N,V,T,e_{\mathrm{IS}}\right)\right]$$

where Ω_*IS*_(*N*, *V*, *T*, *e*_*IS*_) is the number of IS in the PEL with energy *e*_*IS*_. The quantity *F*_*vib*_(*N*, *V*, *T*, *e*_*IS*_) in eq 6 is the vibrational Helmholtz free energy and is defined as

8 $$F_{\mathrm{vib}}\left(N,V,T,e_{\mathrm{IS}}\right)\equiv -k_{B}T\ln\left[\langle Q_{l}\left(N,V,T\right)\rangle_{e_{\mathrm{IS}}}\right]$$

where *Q*_*l*_(*N*, *V*, *T*) is the canonical partition function of the system when it is trapped within the basin *l* of the PEL, i.e.,

9 Ql(N,V,T)=1h3nbn∫−∞∞[∏i=1ndpi1...dpinb]exp[−β∑i=1n∑k=1nb(pik)22mi′]∫Vl[∏i=1ndri1...drinb]exp[−βΔURP(R)]

Here, $\Delta U_{\mathrm{RP}}\left(R\right)$ is the potential energy of the system relative to the basin IS energy, *e*_*IS*_ ( $\Delta U_{\mathrm{RP}}=U_{\mathrm{RP}}-e_{\mathrm{IS}}$ ). In eq 8, ⟨··· ⟩_*e*_*IS*__ indicates averaging over all basins *l* of the PEL with IS energy *e*_*IS*_. As shown below, *F*_*vib*_(*N*, *V*, *T*, *e*_*IS*_) is the contribution to the Helmholtz free energy arising from the exploration by the system of the PEL basins with energy *e*_*IS*_.

Equation 6 is exact, but it is of no practical use. As discussed extensively in the literature,^32–34,45^ in the PEL formalism, one assumes that the system in equilibrium samples a narrow range of *e*_*IS*_-values, which is consistent with numerous computational studies,^46,47^ as long as the system remains in only one-phase.^48,49^ Equivalently, one applies a saddle-point approximation in eq 6, resulting in the following expression

10 $$Q\left(N,V,T\right)\approx e^{-\beta \left[E_{\mathrm{IS}}-TS_{\mathrm{IS}}\left(N,V,T,E_{\mathrm{IS}}\right)+F_{\mathrm{vib}}\left(N,V,T,E_{\mathrm{IS}}\right)\right]}$$

In eq 10, *E*_*IS*_ = *E*_*IS*_(*N*, *V*, *T*) is the IS energy that maximizes the argument of the sum in eq 6 and is the solution of

$$1-T{\left(\frac{\partial S_{\mathrm{IS}}\left(N,V,T,e_{\mathrm{IS}}\right)}{\partial e_{\mathrm{IS}}}\right)}_{N,V,T}+{\left(\frac{\partial F_{\mathrm{vib}}\left(N,V,T,e_{\mathrm{IS}}\right)}{\partial e_{\mathrm{IS}}}\right)}_{N,V,T}=0\;\left(\mathrm{with}\;e_{\mathrm{IS}}\to E_{\mathrm{IS}}\right)$$ 11

In computational studies, *E*_*IS*_(*N*, *V*, *T*) is identified with the average IS energy sampled by the system at the given (*N*, *V*, *T*), i.e., *E*_*IS*_(*N*, *V*, *T*) = ⟨*e*_*IS*_ ⟩_*N*, *V*, *T*_.

### 2.1 Gaussian and Harmonic Approximation of the PEL

In order to proceed further within the PEL formalism, one must model the statistical properties of the PEL. Specifically, one needs to introduce an ansatz for *S*_*IS*_ and *F*_*vib*_; see eqs 7 and 8. The two most common and well-tested approximations in the PEL formalism (see refs 32–34,45,47,50–54 ), are the (i) Gaussian and (ii) harmonic approximations.(i) In the Gaussian approximation of the PEL, it is assumed that

12 $$\Omega_{\mathrm{IS}}\left(N,V,T,e_{\mathrm{IS}}\right)=\frac{1}{\sqrt{2\pi \sigma^{2}}}e^{\alpha N}e^{-{\left(e_{\mathrm{IS}}-E_{0}\right)}^{2}/2\sigma^{2}}$$

where (α, σ^2^, *E*_0_) are PEL-variables that, in principle, depend on (*V*, *T*). Using this expression in eq 7, one finds that

13 $$S_{\mathrm{IS}}\left(N,V,T,e_{\mathrm{IS}}\right)\approx k_{B}\left[\alpha N-\frac{{\left(e_{\mathrm{IS}}-E_{0}\right)}^{2}}{2\sigma^{2}}\right]$$

(ii) In the harmonic approximation (HA) of the PEL, it is assumed that the basins of the PEL about the IS are quadratic (harmonic). This allows one to evaluate eq 9 analytically. It can be shown from eq 8 that under the HA of the PEL^33,36,37^

$$F_{\mathrm{vib}}\left(N,V,T,e_{\mathrm{IS}}\right)\equiv F_{\mathrm{vib}}^{\mathrm{harm}}\left(N,V,T,e_{\mathrm{IS}}\right)=dn_{b}k_{B}T\ln\left(\beta A_{0}\right)+k_{B}TS\left(N,V,T,e_{\mathrm{IS}}\right)$$ 14

where *d* = 9*N* is the number of degrees of freedom of the atomistic water model system, and $S\left(N,V,T,e_{\mathrm{IS}}\right)$ is the basin shape function.^33,36^ $S\left(N,V,T,e_{\mathrm{IS}}\right)$ quantifies the local curvature of the PEL basins with energy *e*_*IS*_ about the corresponding IS. Specifically

$$S\left(N,V,T,e_{\mathrm{IS}}\right)={\left\langle \overset{dn_{b}-3}{\underset{i=1}{\sum }}\ln\left(\frac{\hbar \omega_{i}\left(N,V,T\right)}{A_{0}}\right)\right\rangle }_{e_{\mathrm{IS}}}$$ 15

In this expression, {ω_*i*_^2^}_*i*=1,2,..._ are the eigenvalues of the *mass-weighted* Hessian matrix of the ring-polymer system evaluated at the IS of the PEL. The ⟨···⟩_*e*_*IS*__ indicates an averaging over all IS of the PEL with energy *e*_*IS*_. Note that since the PEL of the quantum liquid is *T*-dependent (eq 5), the quantities {ω_*i*_^2^}_*i*=1,2,..._ also vary with *T*. In eq 15, *A*_0_ ≡ 1 kJ/mol is a constant that ensures that the argument of ln(···) has no units. Since an IS is a potential energy minimum, all the *dn*_*b*_ eigenvalues of the *mass-weighted* Hessian matrix are greater than zero, except for 3 eigenvalues associated with the system’s center of mass motion which are zero. As will be shown below, and consistent with previous path-integral computer simulations,^36–38^ we find that for all the water isotopes studied

$$S\left(N,V,T,e_{\mathrm{IS}}\right)=a\left(N,V,T\right)+b(N,V,T)\;e_{\mathrm{IS}}$$ 16

where *a* and *b* are coefficients that depend on (*N*, *V*, *T*).

With the Gaussian and harmonic approximation of the PEL (eqs 13 and 14), one can write the Helmholtz free energy of the system, *F*(*N*, *V*, *T*) = −*k*_*B*_ *T *ln­[*Q*(*N*, *V*, *T*)]. Specifically, from eq 10

17 $$F\left(N,V,T\right)=E_{\mathrm{IS}}\left(N,V,T\right)-TS_{\mathrm{IS}}\left(N,V,T,E_{\mathrm{IS}}\right)+F_{\mathrm{vib}}\left(N,V,T,E_{\mathrm{IS}}\right)$$

Similarly, from eqs 11, 13, and 14, the IS energy is given by^36,37^

18 $$E_{\mathrm{IS}}\left(N,V,T\right)\equiv E_{\mathrm{IS}}^{\mathrm{harm}}\left(N,V,T\right)=E_{0}-\sigma^{2}\left(\beta +b\right)$$

where, $b\equiv {\left(\frac{\partial S\left(N,V,T,E_{\mathrm{IS}}\right)}{\partial E_{\mathrm{IS}}}\right)}_{N,V,T}$ .

All thermodynamic properties follow from eq 17. For example, from eqs 17 and 18, it can be shown that the total energy of the system, *E*(*N*, *V*, *T*) = (∂(*βF*)/∂β)_*N*,*V*_ is given by *E* = *E*_*IS*_ + *E*_*vib*_ where

19 $$E_{\mathrm{vib}}\left(N,V,T\right)\equiv E_{\mathrm{vib}}^{\mathrm{harm}}\left(N,V,T\right)=dn_{b}k_{B}T+{\left(\frac{\partial S}{\partial \beta }\right)}_{N,V,E_{\mathrm{IS}}}$$

is the vibrational energy of the system. Similarly, the entropy of the system *S*(*N*,*V*,*T*) is given by *S* = *S*_*IS*_ + *S*_*vib*_, where

20 $$S_{\mathrm{vib}}\left(N,V,T\right)\equiv S_{\mathrm{vib}}^{\mathrm{harm}}\left(N,V,T\right)=dn_{b}k_{B}\left[1-\ln\left(\beta A_{0}\right)\right]-k_{B}S+k_{B}\beta {\left(\frac{\partial S}{\partial \beta }\right)}_{N,V,E_{\mathrm{IS}}}$$

In eqs 18–20, we have assumed that the PEL variables *E*_0_ = *E*_0_(*V*) and σ^2^ = σ^2^(*V*) (i.e., they are *T*-independent), and that *b* = *b*(*N*, *V*, *T*) [see eq 16 and refs 36,37 ]. Note that in the PEL formalism for a classical liquid, *E*_0_, σ^2^, *b*, and S are necessarily *T*-independent since the PEL of classical liquids cannot depend on *T*. In this regard, eqs 19 and 20 reduce to the expressions obtained for classical (q-TIP4P/F) water in ref 39, *E*_*vib*_^*harm*^ = *dk*_*B*_ *T* and $S_{\mathrm{vib}}^{\mathrm{harm}}={\mathrm{dk}}_{B}\left[1-\ln\left(\beta A_{0}\right)\right]-k_{B}S$ .

### 2.2 Anharmonic Corrections to the PEL Formalism for Quantum Water

Previous classical PEL studies of rigid and flexible water models such as SPC/E, TIP4*P*/2005, and q-TIP4P/F show that the HA of the PEL does not hold. In these cases, one needs to introduce anharmonic corrections. Next, we describe how anharmonic corrections can be modeled in the PEL formalism for quantum water. We follow the approach introduced in ref 38 for the case of a quantum atomistic binary mixture liquid. While anharmonic contributions are assumed to depend on *T* and *e*_*IS*_, as we will show in this work, the anharmonic contributions for q-TIP4P/F water depend only on *T*.

In order to include anharmonic corrections in the PEL formalism, the vibrational Helmholtz free energy of the system is expressed as

21 $$F_{\mathrm{vib}}\left(N,V,T,e_{\mathrm{IS}}\right)=F_{\mathrm{vib}}^{\mathrm{harm}}\left(N,V,T,e_{\mathrm{IS}}\right)+F_{\mathrm{vib}}^{\mathrm{anh}}\left(N,V,T,e_{\mathrm{IS}}\right)$$

where *F*_*vib*_^*harm*^(*N*, *V*, *T*, *e*_*IS*_) is given by eq 14 and *F*_*vib*_^*anh*^(*N*, *V*, *T*, *e*_*IS*_) is the correction due to the basins anharmoncitiy. Note that we write *F*_*vib*_^*anh*^ as an explicit function of *e*_*IS*_ and hence, eq 21 is general, with no underlying assumptions. Substituting eqs 13 and 21 into eq 11 leads to the following expression for the IS energy

22 $$E_{\mathrm{IS}}\left(N,V,T\right)=E_{\mathrm{IS}}^{\mathrm{harm}}\left(N,V,T\right)+E_{\mathrm{IS}}^{\mathrm{anh}}\left(N,V,T,E_{\mathrm{IS}}\right)$$

where *E*_*IS*_^*harm*^(*N*, *V*, *T*) is given by eq 18 and

23 $$E_{\mathrm{IS}}^{\mathrm{anh}}\left(N,V,T,E_{\mathrm{IS}}\right)=-\sigma^{2}{\left(\frac{\partial \left(\beta F_{\mathrm{vib}}^{\mathrm{anh}}\left(N,V,T,E_{\mathrm{IS}}\right)\right)}{\partial E_{\mathrm{IS}}}\right)}_{N,V,T}$$

The new expression for *E*_*vib*_ ≡ *E* – *E*_*IS*_ follows from the thermodynamic relation *E* = (∂(*βF*)/∂β)_*N*,*V*_ and eqs 22 and 23 (see ref 38 for details). Specifically, one obtains

24 $$E_{\mathrm{vib}}\left(N,V,T\right)=E_{\mathrm{vib}}^{\mathrm{harm}}\left(N,V,T\right)+E_{\mathrm{vib}}^{\mathrm{anh}}\left(N,V,T,E_{\mathrm{IS}}\right)$$

where *E*_*vib*_^*harm*^(*N*, *V*, *T*) is given by eq 19 and

25 $$E_{\mathrm{vib}}^{\mathrm{anh}}\left(N,V,T,E_{\mathrm{IS}}\right)={\left(\frac{\partial \left(\beta F_{\mathrm{vib}}^{\mathrm{anh}}\left(N,V,T,E_{\mathrm{IS}}\right)\right)}{\partial \beta }\right)}_{N,V,E_{\mathrm{IS}}}$$

Similarly, a new expression for *S*_*vib*_ follows from the thermodynamic relation *S* = *k*_*B*_β^2^(∂*F*/∂β)_*N*, *V*_. It can be shown that *S*(*N*, *V*, *T*) = *S*_*IS*_(*N*, *V*, *T*, *E*_*IS*_) + *S*_*vib*_(*N*, *V*, *T*), where

26 $$S_{\mathrm{vib}}\left(N,V,T\right)=S_{\mathrm{vib}}^{\mathrm{harm}}\left(N,V,T\right)+S_{\mathrm{vib}}^{\mathrm{anh}}\left(N,V,T,E_{\mathrm{IS}}\right)$$

In this expression, *S*_*vib*_^*harm*^(*N*, *V*, *T*) is given by eq 20 and

$$S_{\mathrm{vib}}^{\mathrm{anh}}\left(N,V,T,E_{\mathrm{IS}}\right)=k_{B}\beta^{2}{\left(\frac{\partial F_{\mathrm{vib}}^{\mathrm{anh}}\left(N,V,T,E_{\mathrm{IS}}\right)}{\partial \beta }\right)}_{N,V,E_{\mathrm{IS}}}$$ 27

In this work, we follow ref 38 and assume that the anharmonic corrections for the case of water can be modeled using the following expression

$$\beta F_{\mathrm{vib}}^{\mathrm{anh}}\left(N,V,T,e_{\mathrm{IS}}\right)={\mathrm{B̃}}_{0}\left(N,V,T\right)+{\mathrm{B̃}}_{1}\left(N,V,T\right)e_{\mathrm{IS}}$$ 28

where *B̃*_0_ and *B̃*_1_ are coefficients that depend on (*N*, *V*, *T*) [and *e*_*IS*_ ≡ *E*_*IS*_ in equilibrium]. Using eq 28 into eqs 23, 25, and 27 one obtains the following expressions

$$E_{\mathrm{IS}}^{\mathrm{anh}}\left(N,V,T,E_{\mathrm{IS}}\right)=-\sigma^{2}{\mathrm{B̃}}_{1}$$ 29

$$E_{\mathrm{vib}}^{\mathrm{anh}}\left(N,V,T,E_{\mathrm{IS}}\right)={\left(\frac{\partial {\mathrm{B̃}}_{0}}{\partial \beta }\right)}_{N,V}+{\left(\frac{\partial {\mathrm{B̃}}_{1}}{\partial \beta }\right)}_{N,V}E_{\mathrm{IS}}$$ 30

and

31 $$S_{\mathrm{vib}}^{\mathrm{anh}}\left(N,V,T,E_{\mathrm{IS}}\right)=k_{B}\left[-\left({\mathrm{B̃}}_{0}+{\mathrm{B̃}}_{1}E_{\mathrm{IS}}\right)+\beta {\left(\frac{\partial {\mathrm{B̃}}_{0}}{\partial \beta }\right)}_{N,V}+\beta {\left(\frac{\partial {\mathrm{B̃}}_{1}}{\partial \beta }\right)}_{N,V}E_{\mathrm{IS}}\right]$$

To summarize, the prediction of the PEL formalism for water, under the Gaussian approximation and assuming anharmonicites of the form given by eq 28, are as follows

$$F_{\mathrm{vib}}\left(N,V,T,E_{\mathrm{IS}}\right)=dn_{b}k_{B}T\ln\left[\beta A_{0}\right]+k_{B}TS\left(N,V,T,E_{\mathrm{IS}}\right)+\frac{1}{\beta }\left({\mathrm{B̃}}_{0}\left(N,V,T\right)+{\mathrm{B̃}}_{1}\left(N,V,T\right)E_{\mathrm{IS}}\left(N,V,T\right)\right)$$ 32

$$E_{\mathrm{IS}}\left(N,V,T\right)=E_{0}-\sigma^{2}\left(\beta +b\left(N,V,T\right)+{\mathrm{B̃}}_{1}\left(N,V,T\right)\right)$$ 33

$$E_{\mathrm{vib}}\left(N,V,T\right)=dn_{b}k_{B}T+{\left(\frac{\partial S}{\partial \beta }\right)}_{N,V,E_{\mathrm{IS}}}+{\left(\frac{\partial {\mathrm{B̃}}_{0}}{\partial \beta }\right)}_{N,V}+{\left(\frac{\partial {\mathrm{B̃}}_{1}}{\partial \beta }\right)}_{N,V}E_{\mathrm{IS}}\left(N,V,T\right)$$ 34

$$S_{\mathrm{vib}}\left(N,V,T\right)=dn_{b}k_{B}\left[1-\ln\left(\beta A_{0}\right)\right]-k_{B}S+k_{B}\beta {\left(\frac{\partial S}{\partial \beta }\right)}_{N,V,E_{\mathrm{IS}}}+k_{B}\left[-\left({\mathrm{B̃}}_{0}+{\mathrm{B̃}}_{1}E_{\mathrm{IS}}\right)+\beta {\left(\frac{\partial {\mathrm{B̃}}_{0}}{\partial \beta }\right)}_{N,V}+\beta {\left(\frac{\partial {\mathrm{B̃}}_{1}}{\partial \beta }\right)}_{N,V}E_{\mathrm{IS}}\left(N,V,T\right)\right]$$ 35

### 2.3 Calculating the PEL Parameters for q-TIP4P/F Water

The theoretical expressions derived above from the PEL formalism will be used to model the results from path-integral computer simulations of H_2_O, HDO, D_2_O, and T_2_O. In this section, we explain how the PEL parameters {α, σ^2^, *E*_0_, *a*, *b*, *B̃*_0_, *B̃*_1_} are obtained from the simulations. In the rest of this work, we omit the explicit dependence of the PEL variables on (*N*,*V*) since our computer simulations are performed at constant *N* and *V*.

Quantities *a* and *b*: The PEL variables *a*(*T*) and *b*(*T*), for each of the q-TIP4P/F water isotopes studied, are calculated using eq 16. To do so, we evaluate the corresponding basin shape function S as a function of *e*_*IS*_ using eq 15. The 3*nn*_*b*_ normal-mode frequencies of the ring-polymer system are evaluated for a large set of IS of the PEL. We confirm that for all the water isotopes studied, and for all the temperatures considered, the ring-polymers are collapsed at the IS sampled by the system. Hence, as explained in detail in ref 36,37 , the 3nn_b_ vibrational frequencies of the ring-polymer system (associated with the water isotope considered) can be calculated in a straightforward manner by using the following expression

$$\omega_{i,j}^{2}=\frac{\omega_{i,0}^{2}}{n_{b}^{2}}-\frac{2}{{\left(\hbar \beta \right)}^{2}}\left[\cos\left(\frac{2\pi \;j}{n_{b}}\right)-1\right]$$ 36

In this expression *i* = 1,2, ..., 9*N*, *j* = 1,2, ..., *n*_*b*_, and ω_*i*,0_^2^ are the normal-mode frequencies of the *classical* q-TIP4P/F water. Specifically, the quantities {ω_*i*,0_^2^}_*i* = 1,2,...,9*N*_ are the eigenvalues of the mass-weighted Hessian matrix of q-TIP4P/F water obtained from classical MD simulations. To calculate {ω_*i*,0_}_*i* = 1,2,...,9*N*_, we perform classical MD simulations of H_2_O using the q-TIP4P/F model at 200 ≤ *T* ≤ 400 K and at the same volume considered in the path-integral computer simulations. At each temperature, we extract 25 configurations and calculate the corresponding IS. The elements of the Hessian matrix, *H*_α,β_ (α, β = 1,2,...,9*N*), are then evaluated at each of these IS. The elements of the *mass-weighted* Hessian matrix are then, ${\mathrm{H̃}}_{\alpha ,\beta }=\frac{H_{\alpha ,\beta }}{\sqrt{m_{\alpha }m_{\beta }}}$ where *m*_α_ and *m*_β_ are the masses of the atoms (H or O) associated with column α and row β of the Hessian matrix. Evidently, the *mass-weighted* Hessian matrix elements are affected by isotope substitutions via the prefactor $\frac{1}{\sqrt{m_{\alpha }m_{\beta }}}$ , while the Hessian matrix elements, *H*_α_,_β_ are not. *For a given temperature T*, the normal-mode frequencies of HDO, D_2_O, and T_2_O are obtained using eq 36 and then, they are combined in eq 15 to calculate the corresponding basin shape function, $S\left(T,e_{\mathrm{IS}}\right)$ . As shown in ref 36 for the case of H_2_O (see also discussion below), $S\left(e_{\mathrm{IS}}\right)$ obeys eq 16 remarkably well. Hence, by fitting $S\left(T,e_{\mathrm{IS}}\right)$ using eq 16, one can calculate the PEL variables *a* and *b* (*for a given T*).

*Quantities α, E_0_, σ^2^*: Following refs 36–38 , we assume that the PEL variables {α, *E*_0_, σ^2^} are T-independent and hence, they are only functions of *V*. The PEL variables {α, *E*_0_, σ^2^} are important since they define the distribution of IS energies in the PEL of the system considered (eq 12). Accordingly, one may expect that these PEL variables vary among the water isotopes studied. However, as explained in ref 38, the collapse of the ring-polymers at the IS (at all conditions studied) implies that the distribution of IS energies in the RP-PELs of water and its isotopes should be identical to one another. In particular, this distribution of IS energies should also be identical to the distribution of IS energies in the PEL of *classical* water. This is because, if the ring-polymers are always collapsed at the IS, then there is an one-to-one relationship between the IS of classical water (sampled in classical MD simulations) and the IS of quantum water/water isotopes (sampled in path-integral computer simulations); see discussions in refs 36,37 . Therefore, we evaluate the PEL variables {α, *E*_0_, σ^2^} from classical MD simulations of H_2_O using the q-TIP4P/F water model (note that in classical MD simulations, the mass of the water atoms play no role in defining these PEL parameters and hence, they are not sensitive to whether one performs classical MD simulations of H_2_O, D_2_O, or T_2_O). The PEL parameters {α, *E*_0_, σ^2^} are taken from our classical MD simulations of H_2_O in ref 39.

To support these ideas, we also calculate the PEL variables {*E*_0_, σ^2^} directly from the path-integral computer simulations of the water isotopes. This is done by first evaluating *E*_*IS*_(*T*) at different temperatures using PIMD simulations, and then fitting *E*_*IS*_(*T*) using eq 18; see ref 36 As shown below, the values of {*E*_0_, σ^2^} from the path-integral computer simulations of H_2_O, HDO, D_2_O and T_2_O practically coincide with the corresponding values of {*E*_0_, σ^2^} obtained from classical MD simulations.

*Quantities B̃_0_ and B̃_1_*: To evaluate these quantities, we follow the procedure employed in ref 38. To calculate *B̃*_1_, we first evaluate *E*_*IS*_^*anh*^(*T*) using eq 22 where *E*_*IS*_(*T*) is obtained directly from the PIMD simulations of water, and *E*_*IS*_^*harm*^(*T*) is given by eq 18. *B̃*_1_ is evaluated from the so-obtained *E*_*IS*_^*anh*^ using eq 29. As we show below, we find that at the temperatures of interest, *B̃*_1_ ≈ 0. A similar procedure is followed to evaluate *B̃*_0_. First, we calculate *E*_*vib*_^*anh*^(*T*) using eq 24 where *E*_*vib*_(*T*) = *E*(*T*)– *E*_*IS*_(*T*) is obtained directly from the path-integral computer simulations of water, and *E*_*vib*_^*harm*^(*T*) is given by eq 19. *B̃*_0_ follows by fitting the so-obtained *E*_*vib*_^*anh*^ using eq 30 with *B̃*_1_ = 0. We find that for the water isotopes studied, the expansion

37 $${\mathrm{B̃}}_{0}=c_{0,0}+c_{0,1}T+c_{0,2}T^{2}$$

­(where {*c*_0,*j*_}_*j* = 0,1,2_ are constants) fits *E*_*vib*_^*anh*^(*T*) very well. Strictly speaking, the procedure described above provides *c*_0,1_ and *c*_0,2_ but cannot be used to calculate *c*_0,0_ because of the partial derivative (∂*B̃*_0_/∂β)_*N*,*V*_ in eq 30. As explained below, the coefficient *c*_0,0_ is obtained by evaluating the entropy *S*(*T*) (via thermodynamic integration^55^) and using eqs 13 and 35.

## 3 Simulation Method

Our results are based on constant-temperature ring-polymer molecular dynamics (RPMD) simulations of H_2_O, HDO, D_2_O, and T_2_O using the q-TIP4P/F water model.^56^ RPMD reduces to PIMD when calculating structural and thermodynamic properties. When considering dynamical properties, such as the diffusion coefficients, RPMD (with or without a thermostat) provides approximate results. In this work, all dynamical properties (diffusion coefficients) are calculated from thermostated RPMD, following the same approach as in refs 18,57,58 . In the case of HDO, all water molecules are modeled as singly deuterated, i.e., consisting of one hydrogen and one deuterium atom per molecule (100% HDO). We note that this is a model system that is not experimentally realizable due to rapid H/D exchange in liquid water,^59^ but it provides a useful intermediate model system, between H_2_O and D_2_O, for the study of isotope substitution effects on the PEL of water. The q-TIP4P/F model is a flexible model, where the intramolecular interaction for the O–H bond is represented with a fourth order polynomial expansion of a Morse potential and a simple harmonic potential is used for the HOH angle.^56^

Our computer simulations are based on a cubic system composed of *N* = 512 water molecules with periodic boundary conditions. The same computational techniques employed in our previous studies are used here (see refs 18,36 for more details). Briefly, we perform RPMD simulations using the OpenMM (version 7.4.0) software package^60^ at constant volume (*v* = 18.0 cm^3^/mol; corresponding to a density for H_2_O of ρ = 1.0 g/cm^3^) and over a wide range of temperatures, 200 ≤ *T* ≤ 400 K. The temperature of the system is controlled using a stochastic (local) path-integral Langevin equation (PILE) thermostat,^61^ where the thermostat collision frequency parameter is set to γ = 0.1 ps^–1^. Short-range (Lennard-Jones pair potential) interactions are calculated using a cutoff of *r*_*c*_ = 1.0 nm, and the long-range electrostatic interactions are computed using the reaction-field technique^62^ with the same cutoff *r*_*c*_. In the RPMD simulations, the time step is *dt* = 0.25 fs, and the number of beads per ring-polymer/atom is set to *n*_*b*_ = 32. For each temperature, the system is equilibrated for 1–50 ns, followed by a production run, of 1–100 ns (depending on the temperature of the system). For comparison, we also perform classical MD simulations of H_2_O for the same temperatures and volume indicated above; the same computational details apply to the RPMD and MD simulations, except that *dt* = 0.50 fs in the MD simulations.

The PEL analysis is based on a total of 25 IS per temperature. These IS are generated from equally spaced configurations saved during the RPMD/MD run (at each temperature). For each of these configurations, the corresponding IS is obtained by minimizing the potential energy of the ring-polymer/classical water system using the L-BFGS-B algorithm.^63^ The IS energy of the system at the local minima are obtained directly from the minimization algorithm.

Since the ring-polymers of water and its isotopes are collapsed at the IS, we only need to calculate the mass-weighted Hessian matrix of classical water (see Section 2.5) via classical MD simulations. To do so, we run classical MD simulations at *v* = 18.0 cm^3^/mol and 200 ≤ *T* ≤ 400 K. At a given *T*, we extract 25 configurations equally spaced in time and evaluate the corresponding IS as explained above. The mass-weighted Hessian matrix at a given IS is calculated analytically. Specifically, for each element of the mass-weighted Hessian, we obtained an analytical expression for the elements of the mass-weighted Hessian based on the q-TIP4P/F pair potential function. This expression is then evaluated using the atoms coordinates at the IS of interest. The mass-weighted Hessian matrix is then diagonalized numerically to obtain the corresponding eigenvalues, which provide the normal-mode frequencies of *classical* q-TIP4P/F water. The curvature of the quantum PEL basins about the corresponding IS for the isotopes of q-TIP4P/F water is obtained by using the same method as in ref 36, eq 36; see Section 2.5.

## 4 Results

The results are organized as follows. In Secs. 4.1, we compare the IS energy *E*_*IS*_(*T*) and vibrational energy *E*_*vib*_(*T*) of H_2_O, HDO, D_2_O, and T_2_O as a function of temperature. Section 4.2 focuses on the atoms delocalization in water and its isotopes, and show that the ring-polymers associated with the O/H/D/T atoms are collapsed at the IS of the corresponding PEL. This allows one to calculate in a straightforward manner the IS vibrational density of states of the ring-polymer system associated to H_2_O, HDO, D_2_O, and T_2_O (and the corresponding shape functions); Section 4.3. In Section 4.4 and 4.5, we test whether the PEL of H_2_O, HDO, D_2_O, and T_2_O are Gaussian and harmonic, respectively. In Section 4.6 and 4.7, we compare the configurational entropy and Kauzmann temperature of H_2_O, HDO, D_2_O, and T_2_O. The configurational entropy of water and its isotopes are used in Section 4.8 to show that the Adam–Gibbs relationship is valid in all cases studied.

### 4.1 Inherent Structure and Vibrational Energy

Figure 1 shows the (a) total energy *E*(*T*), (b) IS energy *E*_*IS*_(*T*), and (c) vibrational energy *E*_*vib*_(*T*) ≡ *E*(*T*) – *E*_*IS*_(*T*) of q-TIP4P/F water as a function of temperature obtained from PIMD simulations. For comparison, also included in Figure 1 are the *E*(*T*), *E*_*IS*_(*T*), and *E*_*vib*_(*T*) of H_2_O obtained from classical MD simulations of q-TIP4P/F water reported in ref 39 (the energy of water obtained from classical MD are identical for all water isotopes since the atoms mass play no role in the thermodynamic properties of classical liquids^64^).

**Figure 1:**
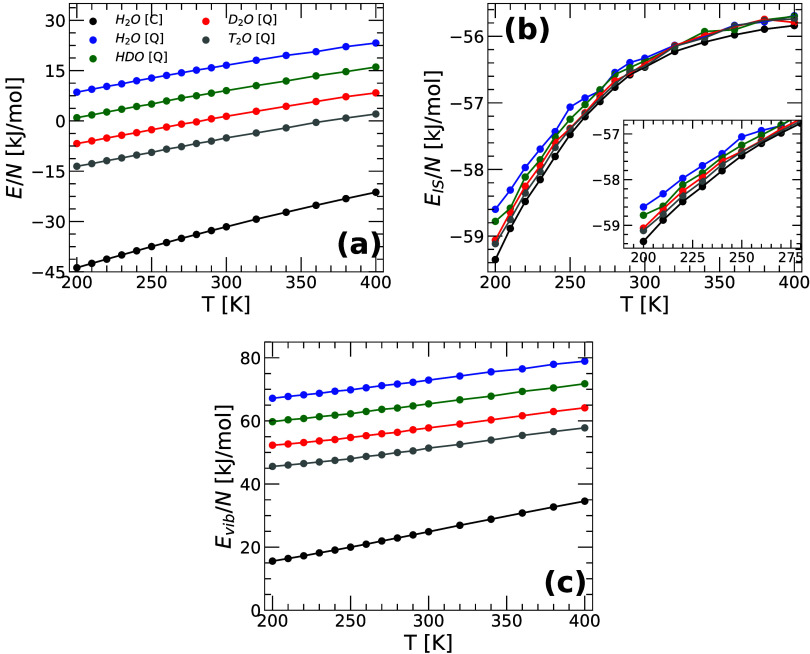
(a) Total energy *E*(*T*), (b) inherent structure energy *E*_*IS*_(*T*), and (c) vibrational energy *E*_*vib*_(*T*) ≡ *E*(*T*) – *E*_*IS*_(*T*) as a function of temperature for the studied isotopes of q-TIP4P/F water (*v* = 18.0 cm^3^/mol). Results for H_2_O, HDO, D_2_O, and T_2_O are indicated by blue, green, red, and gray circles, respectively. For comparison, results obtained from classical MD simulations of H_2_O from ref 39 are also included (black circles). The inset in (b) is a magnification of the main panel for *T* ≤ 300 K. Panel (a) is reproduced from ref 18. Copyright 2025 American Chemical Society.

The energy *E*(*T*) of the different isotopes of q-TIP4P/F water decrease during cooling. Moreover, at a given temperature, *E*(*T*) increases along the sequence T_2_O → D_2_O → HDO → H_2_O, i.e., as the NQE become more pronounced. This is expected since PIMD simulations incorporate NQE, such as zero-point energy, which are relevant, and vary with the mass of the water atoms. Indeed, the energy associated with the bending and stretching modes increase as the mass of the atoms H/D/T covalent-bonded to the oxygen atom decreases. For the same reason, the *E*(*T*) of H_2_O obtained from PIMD simulations (NQE included) is larger than in MD simulations (NQE excluded). We note that total energy differences among the water isotopes are not negligible. For example, the difference in total energy between H_2_O and T_2_O (per molecule) is Δ*E* ≈ 22 kJ/mol which is comparable to the energy associated with a hydrogen-bond.^65^

Figure 1(b) shows the IS energy of H_2_O, HDO, D_2_O, and T_2_O as a function of temperature. Consistent with previous PEL studies of classical^39,50,66,67^ and quantum^36^ water, *E*_*IS*_(*T*) decreases rapidly upon cooling (at *T* < 300 K) while it approaches a constant value at very high temperatures (see, e.g., refs 33,35,68 ). Interestingly, the values of *E*_*IS*_(*T*) vary weakly among the water isotopes studied, within an interval Δ*E*_*IS*_ ≈ 0.50 kJ/mol. At a given temperature (*T* ≤ 300 K), *E*_*IS*_(*T*) increases along the sequence H_2_O­(classical) → T_2_O → D_2_O → HDO → H_2_O (quantum) i.e., as the NQE become more pronounced. This implies that, at a given temperature, the lighter water isotopes visit (slightly) higher regions of the PEL than the heavier isotopes.

The vibrational energy *E*_*vib*_(*T*) for H_2_O, HDO, D_2_O, and T_2_O are shown in Figure 1(c) and follow the same trend observed in Figure 1(a) for the corresponding *E*(*T*). This is a consequence of the close values of *E*_*IS*_(*T*) among the water isotopes [Figure 1(b)], since *E*_*vib*_(*T*) ≡ *E*(*T*) – *E*_*IS*_(*T*). Accordingly, at a given temperature, *E*_*vib*_(*T*) increases as the water isotope becomes lighter, and the NQE become more pronounced.

### 4.2 Atoms Delocalization

The quantum delocalization of the water atoms can be quantified by the spread of the corresponding ring-polymers in the system. Accordingly, we study the delocalization of the O/H/D/T atoms of water by calculating the corresponding radius of gyration *R*_*g*_ given by

$$R_{g}^{2}=\left\langle \frac{1}{n_{b}}\overset{n_{b}}{\underset{k=1}{\sum }}{\left({\mathrm{r⃗}}_{k}-{\mathrm{r⃗}}_{\mathrm{CM}}\right)}^{2}\right\rangle$$ 38

where *r⃗*_*k*_ is the vector position of the *k*-th ring-polymer bead, and *r⃗*_*CM*_ is the center-of-mass of the corresponding ring-polymer; ⟨···⟩ indicates averaging over times and all ring-polymers in the system.

Figure 2(a) shows the values of *R*_*g*_(*T*) obtained from PIMD simulations of H_2_O, HDO, D_2_O, and T_2_O at *v* = 18.0 cm^3^/mol. Consistent with refs 18,22 , the values of *R*_*g*_(*T*) for the O/H/D/T atoms are not negligible, and increase monotonically upon cooling. Interestingly, the delocalization of the O/H/D/T atoms is practically independent of the molecules they belong to. For example, the *R*_*g*_(*T*) of the O atoms is identical in H_2_O, HDO, D_2_O, and T_2_O. Similarly, the *R*_*g*_(*T*) of the H atoms is practically identical in H_2_O and HDO. As expected, at a given temperature, *R*_*g*_(*T*) increases along the sequence T → D → H, i.e., as the atomic mass decreases. Our results in Figure 2(a) are consistent with the fact that the ring-polymer’s spring constants are *k*_*sp*_^*i*^ ∝ *m*_*i*_ *T* ^2^ (eq 5), i.e., as *T* and/or *m*_*i*_ decreases, the spring constants become weaker allowing the beads of the ring-polymers to spread further apart. We also calculate the radius of gyration for the ring-polymers at the IS. Consistent with previous PIMD simulations of H_2_O^36^ and monatomic quantum liquids,^35,37,38^ we find that *R*_*g*_(*T*) < 10^–4^ Å at the IS sampled by the water isotopes. In other words, at all temperatures studied, the ring-polymers associated with the O/H/D/T atoms are collapsed at the IS of the PEL. As an example, included in Figure 2(b) is a snapshot of an HDO molecule at *T* = 200 K taken from an instantaneous configuration and at the corresponding IS.

**Figure 2:**
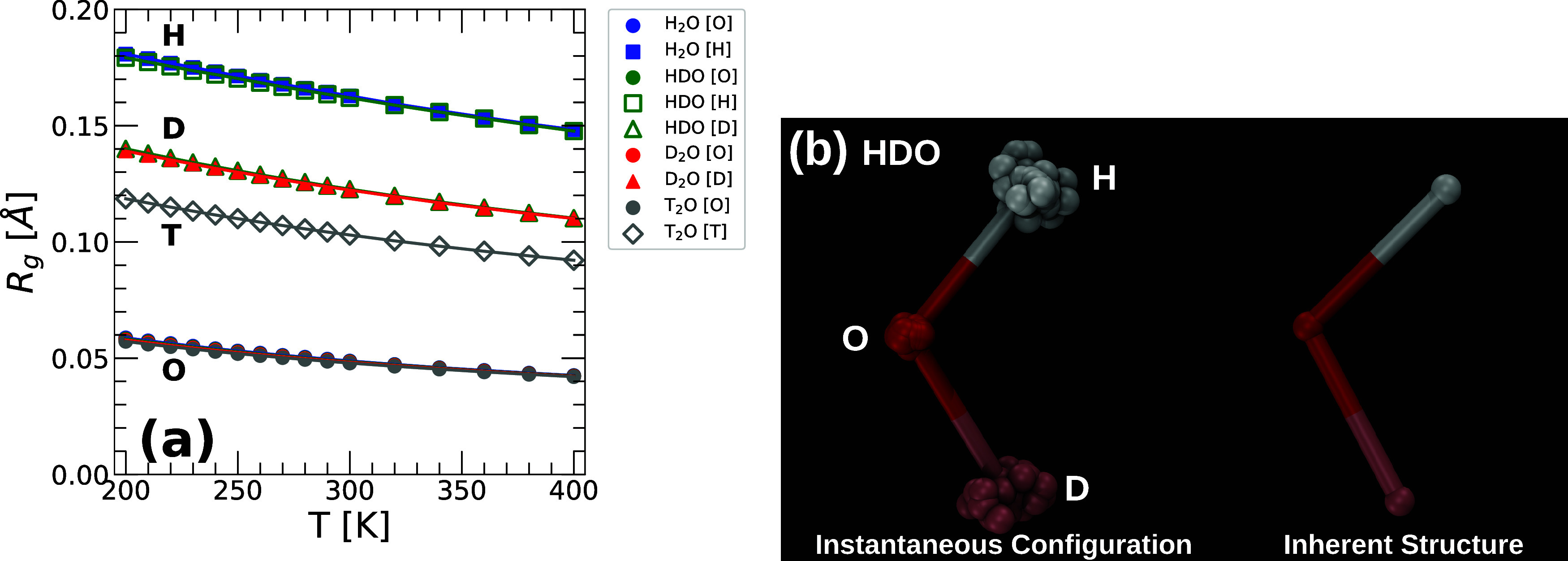
(a) Radius of gyration as a function of temperature, *R*_*g*_(*T*), of the O, H, D, and T atoms of H_2_O, HDO, D_2_O, and T_2_O obtained from PIMD simulations using the q-TIP4P/F water model. Circles, squares, triangles, and diamonds are the *R*_*g*_ of O, H, D, and T, respectively (blue, H_2_O; green, HDO; red, D_2_O; gray T_2_O). (b) Snapshot of an HDO molecule at an instantaneous configuration, and at the corresponding IS (*v* = 18.0 cm^3^/mol and *T* = 200 K); the ring-polymer beads associated with the O, H, and D atoms are shown in red, white, and pink, respectively (*n*_*b*_ = 32). While at the instantaneous configuration the ring-polymers are spread (*R*_*g*_(*T*) > 0), all ring-polymers are collapsed at the corresponding IS (*R*_*g*_(*T*) < 10^–4^ Å).

### 4.3 Inherent Structures Vibrational Density of States and Shape Function

Since the ring-polymers associated with the O/H/D/T atoms are collapsed at the IS of the corresponding RP-PEL, the vibrational frequencies of the ring-polymer system associated with H_2_O, HDO, D_2_O, and T_2_O (at the corresponding IS) can be calculated in a straightforward manner by using eq 36.^36,37^ As discussed in Section 2.5, in eq 36, the vibrational frequencies {ω_*i*,0_ }_*i*=1,2,..9*N*_ of H_2_O are evaluated at the IS of the corresponding classical PEL (CL-PEL; from classical MD simulations).

Figures 3(a) shows the IS vibrational density of states (IS-VDOS) of the ring-polymer system associated with the isotopes of q-TIP4P/F water at (*T* = 240 K, *v* = 18.0 cm^3^/mol). The IS-VDOS is the distribution of normal-mode frequencies of the system, ω_*i*, *j*_ (eq 36), evaluated at the IS sampled by the system at a given working conditions. Consistent with ref 36, the IS-VDOS of the ring-polymer system associated with H_2_O, HDO, D_2_O, and T_2_O [Figure 3(a)] is composed of a large number of peaks that shift toward lower frequencies upon cooling. Interestingly, and consistent with eq 36, the IS-VDOS also shift toward lower frequencies as the NQE become less pronounced, i.e., along the sequence H_2_O→HDO→D_2_O→T_2_O (Figure 3(a)). For comparison, included in Figure 3(b) are the IS-VDOS of the *classical* H_2_O, HDO, D_2_O, and T_2_O [i.e., the distribution of normal-mode frequencies ω_*i*,0_]. As for the quantum cases [Figure 3(a)], Figure 3(b) shows that increasing the mass of the water isotope also shifts the IS-VDOS to lower frequencies.

**Figure 3:**
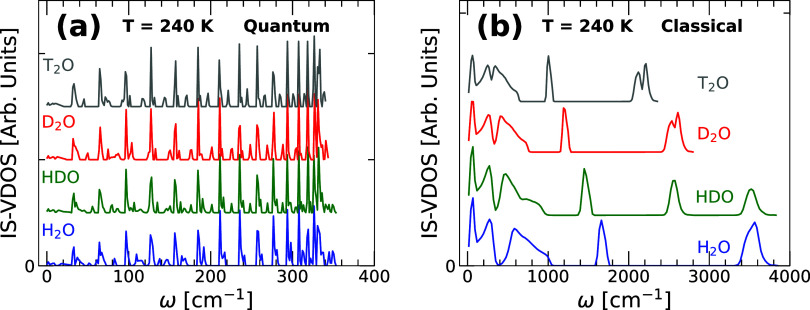
(a) Inherent structures vibrational density of states (IS-VDOS) of the ring-polymer systems associated with H_2_O, HDO, D_2_O, and T_2_O at *T* = 240 K and *v* = 18.0 cm^3^/mol. (b) IS-VDOS of H_2_O, HDO, D_2_O, and T_2_O at *T* = 240 K and *v* = 18.0 cm^3^/mol calculated from classical MD simulations; see text. In both (a) and (b), the IS-VDOS shift toward lower frequencies as the water isotope mass increases. The IS-VDOS in (a) is calculated from the IS-VDOS in (b) using eq 36.

The IS-DOS of the ring-polymer system associated with water and its isotopes [Figure 3(a)] and their classical counterpart [Figure 3(b)] are related via eq 36. The relationship between both IS-VDOS for the case of H_2_O (q-TIP4P/F water) is discussed in detail in our previous work in ref 36; the same discussion/conclusions apply to HDO, D_2_O, and T_2_O. Here, we limit ourselves to stress that only the IS-VDOS of the classical water isotopes [Figure 3(b)] is physically meaningful while the IS-VDOS of the ring-polymer system associated with the water isotopes [Figure 3(a)] is relevant only within the PEL formalism (e.g., to calculate the shape function, see below). The classical IS-VDOS in Figure 3(b) are consistent with experiments. For example, in the case of H_2_O, the IS-VDOS exhibits a stretching frequency band (ω > 3000 cm^–1^), a bending band (ω ≈ 1600 cm^–1^), and translational/librational frequency bands (ω < 1200 cm^–1^). Interestingly, and consistent with experiments,^59,69,70^ all frequency bands shift toward lower frequencies upon the H → D → T substitution. Moreover, along the sequence H_2_O → HDO → D_2_O → T_2_O the translational and librational modes merge with one another, and the stretching band becomes bimodal, suggesting the splitting into two different characteristic stretching modes. Importantly, the results in Figure 3(b) based on the IS-VDOS of the water isotopes are fully consistent with the corresponding IR spectra evaluated from PIMD simulations of (q-TIP4P/F) water reported in ref 18.

Next we discuss the shape function $S\left(N,V,T,e_{\mathrm{IS}}\right)$ for the isotopes of q-TIP4P/F water. The shape function for H_2_O, HDO, D_2_O, and T_2_O is calculated using eq 15 and the IS-VDOS shown in Figure 3(a). Figure 4(a) shows the S for the isotopes of q-TIP4P/F water as a function of temperature at *v* = 18.0 cm^3^/mol. Consistent with previous PIMD simulations of monatomic liquids^35,37^ and H_2_O,^36^ the S(T) of all the water isotopes studied decreases monotonically upon isochoric cooling. This implies that the basins of the RP-PEL sampled by H_2_O, HDO, D_2_O and T_2_O are increasingly wider as the temperature decreases. For comparison, included in the inset of Figure 4(a) are the S(T) values of H_2_O, HDO, D_2_O, and T_2_O obtained from classical MD simulations. Interestingly, classical MD simulations predict the opposite effect of cooling, i.e., the PEL basins of the water isotopes become slightly narrower (larger S(T) ) upon cooling. We note that the values of S(T) for the water isotopes studied are very close to one another and follow within a range of $\Delta S/{\mathrm{Nn}}_{b}\approx 0.8$ from one another. In particular, at a given temperature, S(T) increases along the sequence T_2_O → D_2_O → HDO → H_2_O implying that the basins sampled by water (at a given temperature) are narrower the lighter the isotope mass. This is consistent with eq 15 and the IS-VDOS shown in Figure 3(a) which shift to higher frequencies along the sequence T_2_O → D_2_O → HDO → H_2_O.

**Figure 4:**
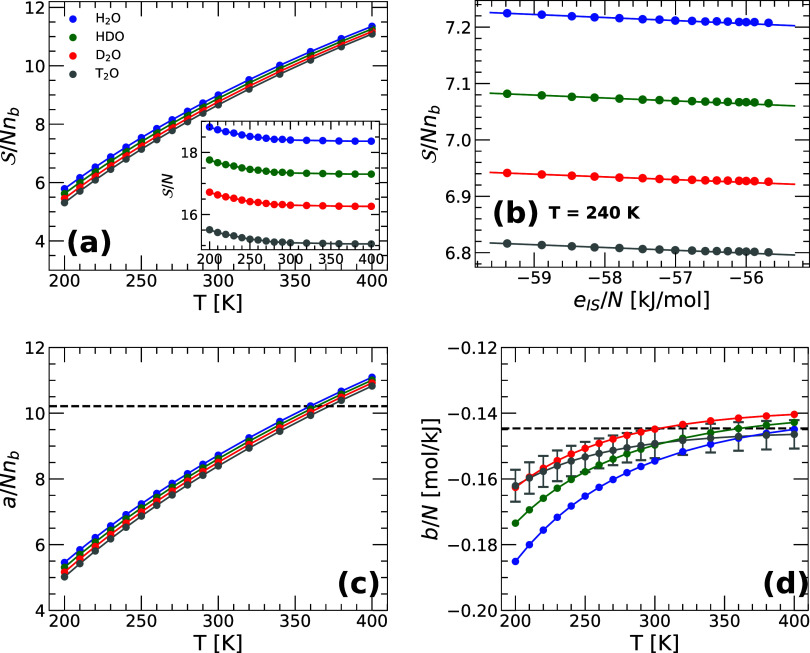
(a) Shape function, S(T) , of the (ring-polymer) PEL associated with H_2_O, HDO, D_2_O, and T_2_O; *v* = 18.0 cm^3^/mol. S(T) is calculated from eq 15 and the IS-VDOS shown in Figure 3(a). Inset: S(T) of H_2_O, HDO, D_2_O, and T_2_O obtained from classical MD simulations. (b) S as a function of *e*_*IS*_ for H_2_O, HDO, D_2_O, and T_2_O at *T* = 240 K and *v* = 18.0 cm^3^/mol. Solid lines are fits using eq 16 (the same procedure employed in ref 36 for the calculation of $S\left(e_{\mathrm{IS}}\right)$ is implemented here). The resulting fitting parameters *a*(*T*) and *b*(*T*) defined in eq 16 are shown in (c) and (d). For comparison, included in (c) and (d) are the (temperature-independent) values of *a* and *b* obtained from classical MD simulations of H_2_O (black dashed lines). In (d), error bars for *b*(*T*) are shown only for the case of T_2_O, as an example.

Previous PIMD simulations show that for quantum liquids, the shape function obeys eq 16. Equation 16 also applies to most classical liquids but, in these cases, *a* = *a*(*N*, *V*) and *b* = *b*(*N*, *V*) since the PEL of a classical liquid is *T*-independent. To show that eq 16 also applies to H_2_O, HDO, D_2_O, and T_2_O, we follow the same procedure of refs 36,37 . Figure 4(b) shows the $S\left(e_{\mathrm{IS}}\right)$ as a function of *e*_*IS*_ for H_2_O, HDO, D_2_O, and T_2_O at *T* = 240 K and *v* = 18.0 cm^3^/mol. It follows that for all water isotopes considered, eq 16 correctly predicts the relationship between the IS energy and the curvatures of the corresponding basin. The coefficients *a*(*T*) and *b*(*T*) are shown in Figure 4(c),4(d); also included are the values of *a* and *b* for classical H_2_O (black dashed line; obtained from classical MD simulations). Interestingly, the PEL variable *b*(*T*) of the (quantum) water isotopes increases monotonically upon heating and seem to approach asymptotically the classical value of *b*; this is not the case of *a*(*T*).

### 4.4 Gaussian Approximation

Next, we show that the Gaussian approximation of the PEL holds for q-TIP4P/F water and its isotopes. Specifically, we test whether the *E*_*IS*_(*T*) of H_2_O, HDO, D_2_O, and T_2_O obtained from the PIMD simulations obeys the prediction of the PEL formalism based on the Gaussian approximation, eq 33. Figure 5(a) shows the *E*_*IS*_(*T*) as a function of *b*(*T*) + β for H_2_O, HDO, D_2_O, and T_2_O obtained from the PIMD simulations (symbols). The lines are the predictions of *E*_*IS*_(*T*) for water and its isotopes based on the Gaussian approximation of the PEL *and* the HA [i.e., *E*_*IS*_(*T*) = *E*_*IS*_^*harm*^(*T*); eq 18]. We note that there are no fitting quantities in eq 18; the PEL variable *b*(*T*) is taken from Figure 4(d) while the constants (*E*_0_,σ^2^) are obtained from the classical MD simulations of q-TIP4P/F water reported in our previous work, ref 39. The anharmonic contributions to *E*_*IS*_ are given by the variable *B̃*_1_(*T*) in eq 29 and can be calculated from Figure 5(a) [−σ^2^ *B̃*_1_ = *E*_*IS*_^*anh*^(*T*) = *E*_*IS*_(*T*) – *E*_*IS*_^*harm*^(*T*) ]. As shown in Figure 5(b), *B̃*_1_(*T*) ≈ 0 up to approximately *T* < 300 K. It follows from eq 28 that, at these temperatures, the vibrational Helmholtz free energy *F*_*vib*_^*anh*^ is only a function of *T* and hence, the basins anharmonicities are independent of *e*_*IS*_ or, equivalently, of the basins location within the PEL. Interestingly, classical MD simulations of water using the SPC/E,^50^ TIP4*P*/2005,^66^ and q-TIP4P/F^39^ models also find that the basins anharmonicities are independent of the corresponding *e*_*IS*_.

**Figure 5:**
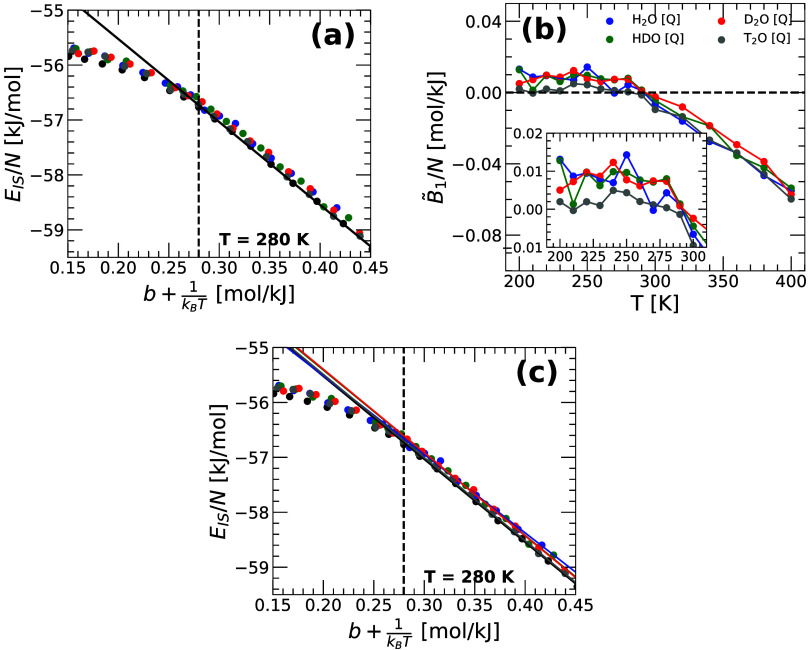
(a) Average IS energy, *E*_*IS*_(*T*), as a function of *b*(*T*) + β for H_2_O, HDO, D_2_O, and T_2_O obtained from PIMD simulations at different temperatures (circles). Lines are the predictions of *E*_*IS*_(*T*) from the PEL formalism based on the Gaussian approximation of the PEL *and* the harmonic approximation, eq 18. The parameters {*E*_0_, σ^2^} used in eq 18 are taken from the classical MD simulations of ref 39. The Gaussian approximation of the PEL, with no anharmonicities, is in very good agreement with the PIMD simulations at approximately *T* ≤ 280 K (vertical dashed line). (b) Anharmonic correction to *E*_*IS*_(*T*) evaluated from (a) using eq 29. For water and its isotopes, *B̃*_1_ ≈ 0 at approximately *T* < 300 K. (c) Same as (a) with the straight fit lines obtained from eq 18 and using *E*_0_ and σ^2^ as free fitting parameters (see Table 1).

One may wonder, how eq 18 may fit the values of *E*_*IS*_(*T*) for water and its isotopes if the parameters {*E*_0_, σ^2^} were not set in advance to match the corresponding values of classical q-TIP4P/F water. To address this question, we include in Figure 5(c) the best fit to the *E*_*IS*_(*T*) values of water and its isotopes (obtained from the PIMD simulations) using eq 18. The resulting fitting parameters {*E*_0_, σ^2^} are given in Table 1. Table 1 shows that, indeed, the values of {*E*_0_, σ^2^} for water and its isotopes obtained from PIMD simulations are practically identical to the corresponding values obtained from classical MD simulations in ref 39.

**Table 1:** Fitting Parameters Resulting from Fig. 5(c) and Using eq 18, for the Cases of H_2_O, HDO, D_2_O, and T_2_O^a^

isotope	*E*_0_ [kJ/mol]	σ^2^ [kJ/mol]^2^
H_2_O [Q]	–52.66 (0.14)	14.30 (0.40)
HDO [Q]	–52.40 (0.12)	15.06 (0.35)
D_2_O [Q]	–52.35 (0.08)	15.16 (0.23)
T_2_O [Q]	–52.46 (0.06)	15.15 (0.18)
H_2_O [C]	–52.51 (0.05)	15.10 (0.15)

a Results are from PIMD simulations [Q]; for comparison also included are the values of (*E*_0_, σ^2^) reported in ref 39 based on classical MD simulations [C]. The standard deviations for the PEL variables *E*_0_ and σ^2^ are shown in parentheses. The PEL parameters (*E*_0_, σ^2^) are practically identical in all cases.

### 4.5 Harmonic Approximation of the PEL and Anharmonic Contributions

In this section, we show that the harmonic approximation of the PEL does not hold for (q-TIP4P/F) water and its isotopes and hence, anharmonic corrections need to be included. This should not be surprising since, even in PEL studies of water using classical MD simulations, anharmonic corrections are necessary.^39,50,66^ Figure 6(a) shows the vibrational energy *E*_*vib*_(*T*) = *E*(*T*) – *E*_*IS*_(*T*) for H_2_O, HDO, D_2_O, and T_2_O obtained from PIMD simulations as a function of temperature at *v* = 18.0 cm^3^/mol [circles; from Figure 1(c)]. The lines are the predictions from the harmonic approximation of the PEL given in eq 19. For comparison, also included is the *E*_*vib*_(*T*) of H_2_O obtained from classical MD simulations (black circles) and the corresponding prediction from the harmonic approximation of the PEL (black line), *E*_*vib*_(*T*) ≡ *E*_*vib*_^*harm*^(*T*) = 9 *Nk*_*B*_ *T*. In all cases, the harmonic approximation of the PEL is in relatively good agreement with the PIMD simulations. However, as shown in Figure 6(b), anharmonic corrections, while small (<1 kJ/mol), are still notable for water and all its isotopes. Indeed the magnitude of *E*_*vib*_^*anh*^(*T*) in Figure 6(b) is comparable to the corresponding corrections reported in previous PEL studies of classical water models.^39,66^ The lines in Figure 6(b) are the fit to *E*_*vib*_^*anh*^(*T*) using eq 30 with the PEL variables *B̃*_1_ = 0 [see discussion above and Figure 5(b)], and *B̃*_0_(*T*) given by eq 37. In all cases, there is an excellent agreement between the PIMD simulation data and eq 30, indicating that anharmonic corrections to the PEL of H_2_O, HDO, D_2_O, and T_2_O can be properly modeled by the anarmonic vibrational free energy given by eqs 28 and 37. Interestingly, Figure 6(b) shows that as one increases the mass of the q-TIP4P/F water isotopes from H_2_O to T_2_O, *E*_*anh*_^*vib*^(*T*) approaches the classical H_2_O value, as one would expect.

**Figure 6:**
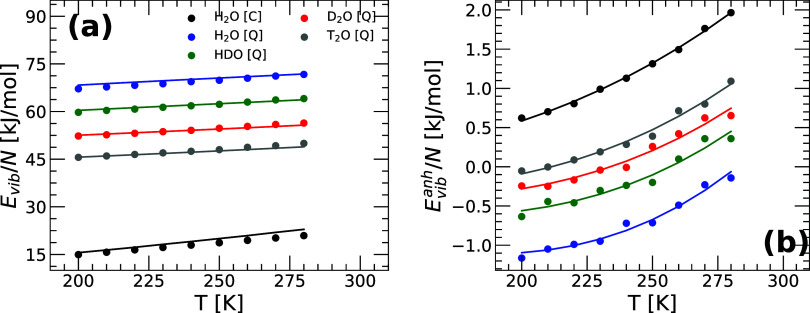
(a) Vibrational energy *E*_*vib*_(*T*) = *E*(*T*) – *E*_*IS*_(*T*) as a function of temperature of the ring-polymer systems associated to water and its isotopes obtained from PIMD simulations of q-TIP4P/F water [circles; from Figure 1(c)]. The solid lines are the theoretical predictions for *E*_*vib*_(*T*) using the harmonic approximation of the PEL, eq 19. (b) The anharmonic vibrational energy *E*_*vib*_^*anh*^(*T*) obtained from (a) [circles]. The lines are the corresponding fit using eq 30 (see text); the fitting parameters {*c*_0,1_, *c*_0,2_} are given in Table 2.

### 4.6 Configurational Entropy

In this section, we show that the configurational entropy *S*_*IS*_(*T*) of H_2_O, HDO, D_2_O, and T_2_O obtained (A) numerically is in agreement with the (B) theoretical expression for *S*_*IS*_(*T*) predicted by the PEL formalism, using the Gaussian approximation [eq 13].

A. *Numerical evaluation of S_IS_(T)*: The numerical calculation of *S*_*IS*_(*T*) is performed by following the same procedure described in ref 38. Briefly, we calculate the configurational entropy from the expression *S*_*IS*_(*T*) = *S*(*T*) – *S*_*vib*_(*T*) where *S*_*vib*_(*T*) is obtained from eqs 20, 26, and 31 or, equivalently, from eq 35, and *S*(*T*) is calculated for H_2_O, HDO, D_2_O, and T_2_O via thermodynamic integration and PIMD simulation at different temperatures and *v* = 18.0 cm^3^/mol (details of these calculations are provided in Appendix A). We note that all quantities in the expression for *S*_*vib*_(*T*) in eq 35 are well-defined except for the constant *c*_0,0_ which is unconstrained. Accordingly, the parameter *c*_0,0_ is obtained by fitting the expression for *S*_*IS*_(*T*) obtained numerically, with the corresponding theoretical expression; see Section (B) below (the parameter *c*_0,0_ for water and its isotopes are given in Table 2).

**Table 2:** Fitting Parameters {*c*_0,0_, *c*_0,1_, *c*_0,2_} Defined in eq 37 ^a^

isotope	*c*_0,0_	*c*_0,1_ [1/K]	*c*_0,2_ [1/K^2^]
H_2_O [Q]	–2.44	0.0113	–1.998 × 10^–5^
HDO [Q]	–1.58	0.0076	–1.485 × 10^–5^
D_2_O [Q]	–1.23	0.0058	–1.244 × 10^–5^
T_2_O [Q]	–0.91	0.0050	–1.181 × 10^–5^
H_2_O [C]	0.00	0.0014	–7.853 × 10^–6^

a *c*_0,1_ and *c*_0.2_ are obtained from the fits shown in Figure 6(b) (solid lines). The parameter *c*_0,0_ is obtained from Figure 7(d) (see text).

Figure 7(a) shows the *S*(*T*) of water and its isotopes (*v* = 18.0 cm^3^/mol). The harmonic and anharmonic contributions to *S*_*vib*_(*T*) [*S*_*vib*_^*harm*^(*T*) from eq 20, and *S*_*vib*_^*anh*^(*T*) from eq 31], are included in Figures 7(b) and 7(c). For comparison, we also include in Figures 7(a)–(c) the *S*(*T*), *S*_*vib*_^*harm*^(*T*), and *S*_*vib*_^*anh*^(*T*) of q-TIP4P/F water reported in ref 39 from classical MD simulations (black circles/lines). The resulting numerical evaluation of *S*_*IS*_(*T*) for water and its isotopes are included in Figure 7(d) (circles).

**Figure 7:**
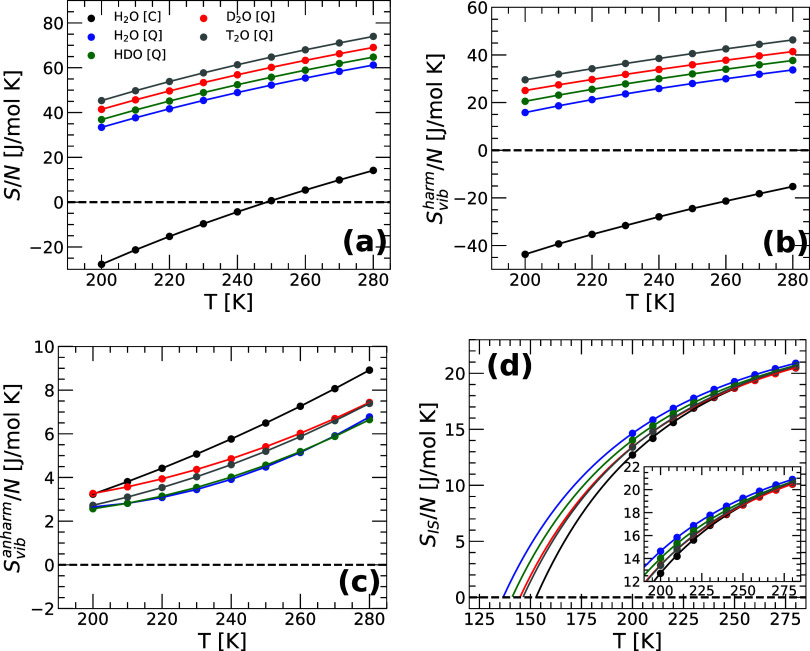
(a) Entropy *S*(*T*) of q-TIP4P/F water and its isotopes as a function of temperature obtained by thermodynamic integrations and PIMD simulations (see Appendix). For comparison, we also include the *S*(*T*) of classical q-TIP4P/F water (black circles/line) reported in ref 39 (obtained by thermodynamic integration and classical MD simulations). Classical MD simulations result in an unphysical negative *S*(*T*) for water while, instead, PIMD simulations (NQE included) result in *S*(*T*) > 0 for water and its isotopes. (b)­(c) Harmonic and anharmonic contributions to the entropy, *S*_*vib*_^*harm*^(*T*) (eq 20) and *S*_*vib*_^*anh*^(*T*) (eq 31) for the same systems included in (a). (d) Configurational entropy, *S*_*IS*_(*T*) = *S*(*T*) – *S*_*vib*_^*harm*^(*T*) – *S*_*vib*_^*anh*^(*T*) obtained numerically from (a), (b), and (c) [circles]. The lines are the PEL formalism prediction for *S*_*IS*_(*T*) from eq 13 [lines]; see text.

B. *Theoretical expression for S_IS_(T)*: The theoretical expression for *S*_*IS*_(*T*) based on the PEL formalism using the Gaussian approximation is given by eq 13. As discussed in Section 2.5 and ref 38, the PEL variables {α, *E*_0_, σ^2^} are taken from the classical MD simulations of ref 39. The lines in Figure 7(d) correspond to the expression of *S*_*IS*_(*E*_*IS*_) from eq 13, after substituting *E*_*IS*_(*T*) using eq 33 [with *B̃*_1_ = 0; see Figure 5(b)]. As shown in Figure 7(d) the agreement between the theoretical expression for *S*_*IS*_(*T*) [lines] and the corresponding numerical calculation [circles] is remarkable. Importantly, at large temperatures, the *S*_*IS*_(*T*) for water and its isotopes (PIMD simulations) all converge to the *S*_*IS*_(*T*) of classical water (MD simulations) reported in ref 39, as one would expect [see also Figure S1 of the Supporting Information (SI)].

The following conclusions also follow from Figure 7. (i) The *S*(*T*) of water and its isotopes increase with increasing temperature which is expected since (∂*S*/∂*T*)_*N*, *V*_ = *C*_*V*_/*T* and the constant volume specific heat for stable liquids is *C*_*V*_ > 0. An interesting point from Figure 7(a) is that *S*(*T*) < 0 for classical water at low temperatures. As discussed in ref 39, this unphysical behavior is a known limitation inherent to classical statistical mechanics and the fact that water has large normal-mode frequencies [specifically, stretching normal-mode frequencies at ω > 3000 cm^–1^; see Figure 3(b)]. Indeed, the *S*(*T*) of water and its isotopes calculated using thermodynamic integration and PIMD simulations are positive at all the temperatures studied [Figure 7(a)].

(ii) The negative/positive values of *S*(*T*) originate in the harmonic contribution to the vibrational entropy, *S*_*vib*_^*harm*^(*T*) (see also ref 39). As shown in Figure 7(b), *S*_*vib*_^*harm*^(*T*) < 0 for classical water (based on MD simulations) but it is positive when NQE are included (using PIMD simulations). Instead, the anharmonic contribution to the vibrational entropy [Figure 7(c)], *S*_*vib*_^*anh*^(*T*), is positive in all cases. Note that the sign of *S*(*T*) is given by the sign of *S*_*vib*_^*harm*^(*T*) since *S*_*vib*_^*anh*^(*T*) represents a small contribution (<10%) to *S*(*T*).

(iii) The total entropy of water and its isotopes (blue, green, red, and gray circles/lines) in Figures 7(a), at a given temperature, increase by up to 15 J/(mol K) (>25%) along the sequence, H_2_O → HDO → D_2_O → T_2_O. This can be understood by noticing that $S_{\mathrm{vib}}^{\mathrm{harm}}\left(T\right)\approx -k_{B}S\left(T\right)$ (eq 20) and the values of S(T) given in Figure 4(a). For example, since S(T) is largest for H_2_O, then H_2_O has the smallest *S*(*T*). In other words, the different IS-VDOS lead to the relative shifts in the *S*(*T*) and *S*_*vib*_^*harm*^(*T*) of water and its isotopes. Note that, compared to the variations in *S*_*vib*_^*harm*^(*T*) [Figure 7(b)], the changes in *S*_*vib*_^*anh*^(*T*) [Figure 7(c)] and *S*_*IS*_(*T*) [Figure 7(d)] among H_2_O, HDO, D_2_O, and T_2_O are relatively small.

### 4.7 Kauzmann Temperature

The Kauzmann temperature, *T*_*K*_, is an important property of liquids/glasses. It is defined as the temperature at which *S*_*IS*_(*T*) = 0.^71^ This means that at *T* ≤ *T*_*K*_, there is only one (amorphous) IS in the PEL available to the system, with the lowest energy. From a practical point of view, *T*_*K*_ represents the lowest possible (kinetic) glass transition temperature of the system. The values of *T*_*K*_ for H_2_O, HDO, D_2_O and T_2_O are obtained numerically from Figure 7(d) as the temperature at which the solid lines intersect the *x*-axis; the values of *T*_*K*_ for water and its isotopes are provided in Table 3. It follows that *T*_*K*_ decreases along the sequence T_2_O → D_2_O → HDO → H_2_O, i.e., as the mass of the isotope decreases. This is consistent with ref 18 where the diffusivity of water also increases along this sequence. The differences in the values of *T*_*K*_ among water and its isotopes are small ≤11 K which is comparable to the *T*-shifts in the thermodynamic properties of H_2_O and D_2_O. For example, the *T*_*K*_ of H_2_O and D_2_O in Table 3 differ by approximately 8 K, close to the difference in the corresponding glass transition temperature *T*_g_ reported in experiments,^72^ ≈ 10 K.

**Table 3:** Kauzmann Temperature *T*_*K*_ for H_2_O, HDO, D_2_O, and T_2_O (at *v* = 18.0 cm^3^/mol) Obtained from Figure 7(d)^a^

Isotope	T_*K*_ [K]
H_2_O [Q]	137
HDO [Q]	141
D_2_O [Q]	145
T_2_O [Q]	146
H_2_O [C]	153

a The value of *T_K_* for classical water is also included [C].

To understand the origin of the isotope substitution effects on *T*_*K*_, we note that

39 $$\frac{1}{T_{K}}=k_{B}\left[\sqrt{\frac{2\alpha N}{\sigma^{2}}}-b\left(T_{K}\right)\right]$$

The expression above follows from eqs 13 and 33 [with *B̃*_1_ = 0, see Figure 5(b)], and the fact that *S*_*IS*_(*T*_*K*_) ≡0. The only quantity in eq 39 that depends on the isotope masses is in the PEL variable *b*(*T*) (evaluated at *T*_*K*_). This is because the parameters {α, *E*_0_, σ^2^}, and hence the corresponding distribution of IS energies in the PEL, are identical for water and its isotopes. Since $b\left(T\right)\equiv {\left(\partial S\left(N,V,T,E_{\mathrm{IS}}\right)/\partial E_{\mathrm{IS}}\right)}_{N,V,T}$ , it follows that the different values of *T*_*K*_ among H_2_O, HDO, D_2_O, and T_2_O are solely due to the different *T*-dependence of the PEL curvature (i.e., the shape function, or, equivalently, the IS-VDOS) of water and its isotopes [Figure 4(d)].

### 4.8 Adam–Gibbs Relationship

Previous MD simulations studies of water^39,73,74^ and other classical liquids^47,75–77^ indicate that the topography of the PEL relates to the dynamics of the system via the Adam–Gibbs (AG) relationship

40 $$D=D_{0}\exp\left[-A/{\mathrm{TS}}_{\mathrm{IS}}\right]$$

where *D* is the diffusion coefficient of the molecules/atoms in the liquid; *D*_0_ and *A* are constants that depend on the system considered. The AG relationship implies that, at a given temperature, the dynamics of the liquid is controlled by the number of available IS in the PEL via the configurational entropy.

Figure 8 shows a semilog plot of *D* as a function of 1/*TS*_*IS*_ at *v* = 18.0 cm^3^/mol for q-TIP4P/F water and its isotopes evaluated from the RPMD simulations (circles) together with the AG prediction, eq 40 (lines); the parameters *A* and *D*_0_ are given in Table 4. It follows that for all the cases considered, the AG relationship is in excellent agreement with our RPMD simulations. We stress that the applicability of the AG relation to water and its isotopes, including NQE, is not evident. Our results imply that while the inclusion of NQE affect the dynamics of water,^18^ the diffusivity of water and its isotopes is still controlled by the topography of the associated RP-PEL.

**Table 4:** Parameters *D*_0_ and *A* Defined in the Adam–Gibbs Relationship, eq 40, and Obtained from the Fittings in Figure 8 for H_2_O, HDO, D_2_O, and T_2_O^a^

isotope	*D*_0_ [Å^2^/ps]	*A* [kJ/mol]
H_2_O [Q]	11.17	–26.16
HDO [Q]	8.05	–24.77
D_2_O [Q]	8.35	–25.39
T_2_O [Q]	9.06	–26.67
H_2_O [C]	11.21	–26.73

a The last row in the table corresponds to the parameters *D*_0_ and *A* obtained from classical MD simulations of q-TIP4P/F water.

**Figure 8:**
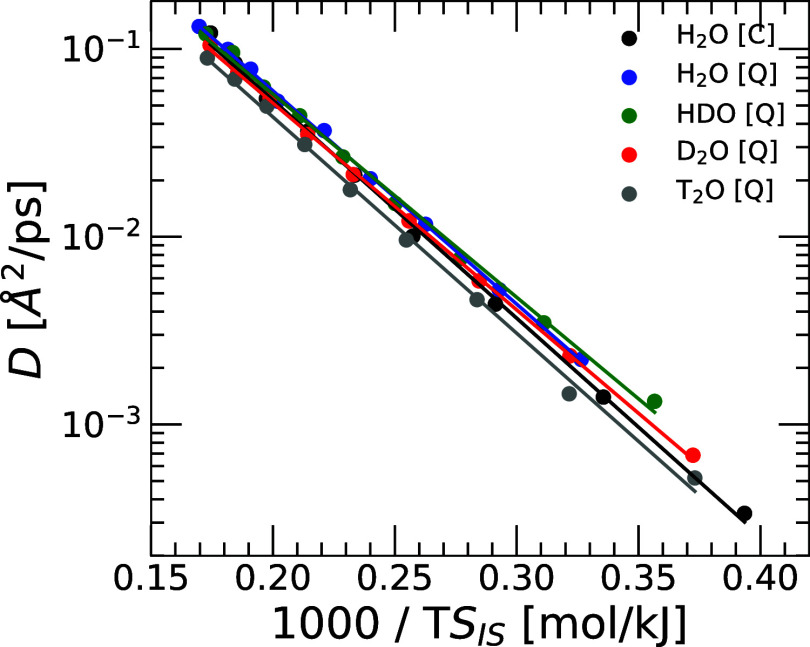
Diffusion coefficient *D*(*T*) as a function of 1000/*TS*_*IS*_ for H_2_O, HDO, D_2_O, and T_2_O at *v* = 18.0 cm^3^/mol. Circles are the *D*(*T*) obtained from RPMD simulations of q-TIP4P/F water at constant temperature; the lines correspond to the best fits obtained using the Adam–Gibbs relationship, eq 40. For comparison, the values of classical H_2_O are also included (black circles/lines) from ref 39. In all cases, irrespective of whether the system obeys classical or quantum mechanics, the Adam–Gibbs relationship works remarkably well for more than 2 orders of magnitude in *D*.

## 5 Summary and Discussion

In this work, we performed path-integral computer simulations of H_2_O, HDO, D_2_O, and T_2_O and presented a detailed characterization of the corresponding RP-PEL at *v* = 18.0 cm^3^/mol. We focused on the *T*-dependence of the main quantities in the PEL formalism; specifically, the IS and vibrational energies [*E*_*IS*_(*T*) and *E*_*vib*_(*T*); Figure 1], IS-VDOS [Figure 3] and shape function [ S(T) ; Figure 4], as well as the vibrational and configurational entropies [*S*_*IS*_(*T*) and *S*_*vib*_(*T*); Figure 7]. By doing so, we were able to identify the topographic features of the PEL of water that are affected by isotope substitution (*H* ↔ *D* ↔ *T*). Our findings can be summarized as follows.

(I) Qualitatively, all the PEL properties of the ring-polymer systems associated with H_2_O, HDO, D_2_O, and T_2_O exhibit a similar behavior with temperature [see, e.g., Figures 1, 2(a), 3(a), 5, 7].

(II) At all temperatures studied, the ring-polymers associated with the O/H/D/T atoms are collapsed at the IS of the corresponding RP-PEL [Figure 2(b)]. This feature has now been observed in a few atomistic and molecular systems^35–37^ and seems to be a common property of the IS of quantum liquids, at least when the atoms delocalization is moderate. It remains unclear, however, whether the ring-polymers may remain spread out at very low temperatures or for very small atomic masses for which the spring forces between the ring-polymer beads become very small (*k*_*sp*_ ∝ *mT* ^2^). From a practical point of view, the collapse of the ring-polymers at the IS of the RP-PEL represents a profound simplification of the PEL formalism since it allows one to calculate the IS-VDOS from classical MD simulations (eq 36), as opposed to PIMD simulations (calculating the IS-VDOS from PIMD simulations is computationally extremely expensive; see ref 36,37 ).

(III) The IS-VDOS of the RP-PEL associated with water and its isotopes are complex [Figure 3(a)] and have little resemblance to the physically relevant VDOS of the liquid [Figure 3(b); see also ref 36]. The IS-VDOS of the ring-polymer system shifts toward higher frequencies along the sequence T_2_O → D_2_O → HDO → H_2_O, i.e, as NQE become more pronounced.

(IV) The Gaussian approximation of the RP-PEL holds for water and its isotopes at approximately *T* < 280–300 K [Figure 5(a)]. This implies that the configurational entropy *S*_*IS*_, of H_2_O, HDO, D_2_O, and T_2_O obey eq 13. Importantly, our results from the PIMD simulations are consistent with the view where the PEL parameters {α, *E*_0_, σ^2^}, and hence *S*_*IS*_(*E*_*IS*_), are identical for H_2_O, HDO, D_2_O, and T_2_O, and equal to the corresponding values for classical water (obtained from classical MD simulations). As we argued in ref 38, this can be rationalized by noticing that the IS of classical water (in its CL-PEL) are isomorphic to the IS of H_2_O, HDO, D_2_O, and T_2_O (in their RP-PEL; see point (II) above).

(V) The harmonic approximation of the PEL formalism does not apply to liquid water (*T* > 200 K). This is consistent with similar findings reported from PEL studies of classical water models.^39,50,66^ Accordingly, anharmonic corrections must be included when the PEL formalism is used to describe H_2_O, HDO, D_2_O, and T_2_O. Fortunately, the anharmonic corrections for water and its isotopes are only *T*-dependent (at *T* < 280 – 300 K) [i.e., *B̃*_1_(*T*) ≈ 0 in eq 33; see also Figure 5(b)].

(VI) The Adam–Gibbs relationship [eq 40], which relates the diffusion coefficient *D*(*T*) of a liquid and its *S*_*IS*_, holds for water and its isotopes [Figure 8]. This implies that the topography of the PEL (*S*_*IS*_) also controls the liquids’ dynamics (*D*) at a given *T*.

The following subtle and important take-home messages follow from this study: (1) The isotope substitution effects in water can be interpreted in very simple topographical terms within the PEL formalism. Specifically, our PIMD simulations are consistent with the view where the IS of water and its isotopes, in their corresponding RP-PEL, are all identical, and isomorphic with the IS of classical water. This implies that the RP-PEL of H_2_O, HDO, D_2_O, and T_2_O are pinned at the *same* IS [see Figure 9] but they differ in other geometrical properties of the PEL, such as the curvature of the basins about the corresponding IS and, most probably, the potential energy barriers separating neighboring IS of the RP-PEL.

**Figure 9:**
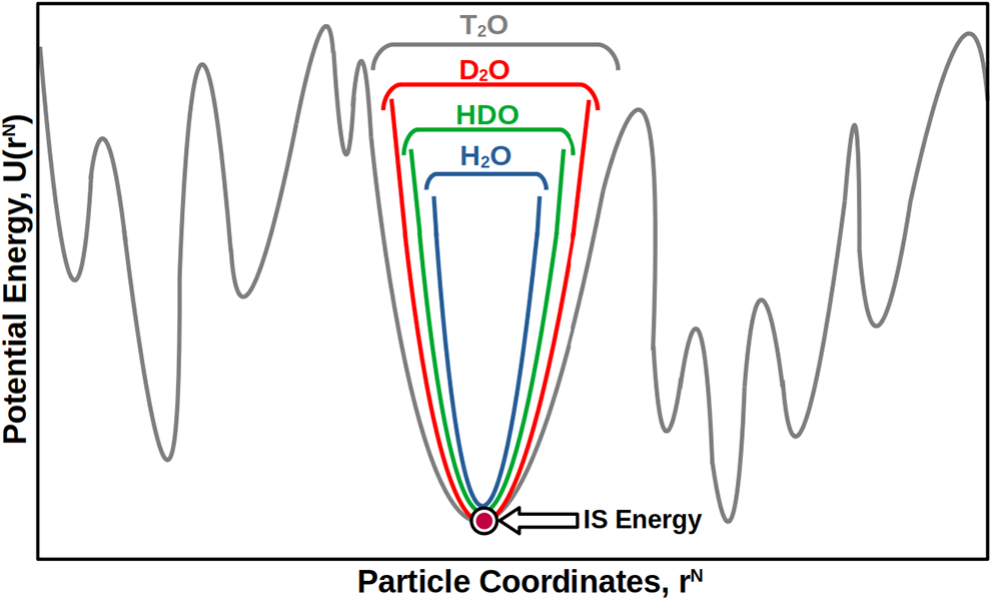
Schematic one-dimensional representation of the RP-PELs associated with H_2_O, HDO, D_2_O, and T_2_O. The RP-PELs of water and its isotopes are pinned at the same IS but they differ in topographic properties, particularly, the basins curvature about the corresponding IS. Basins are narrower as the NQE become more pronounced (along the sequence T_2_O → D_2_O → HDO → H_2_O).

(2) These ideas can be set on precise terms by noticing that the Helmholtz free energy of water and its isotopes, within the PEL formalism, can be written as follows. From eqs 13, 32, and 33 [*B̃*_1_(*T*) = 0]

$$F\left(N,V,T\right)=E_{0}\left(N,V\right)-\sigma^{2}\left(N,V\right)\left[\beta +b\left(N,V,T\right)\right]+dn_{b}k_{B}T\ln\left(\beta A_{0}\right)+k_{B}TS\left(N,V,T,E_{\mathrm{IS}}\right)+\frac{1}{\beta }{\mathrm{B̃}}_{0}\left(N,V,T\right)-TS_{\mathrm{IS}}\left(N,V,T\right)$$ 41

In this expression, the only PEL variables that are affected by isotope substitution are the shape function $S\left(N,V,T,E_{\mathrm{IS}}\right)$ , its partial derivative $b\left(T\right)={\left(\partial S/\partial E_{\mathrm{IS}}\right)}_{N,V,T}$ , and the basins anharmonicity correction *B̃*_0_(*T*). In other words, the only changes in *F*(*N*, *V*, *T*), and hence in all thermodynamic properties of the system, are due to the changes in the basins shape upon the H/D/T substitution in water.

(3) Equation 41 implies that it is possible to quantify the shifts in the different thermodynamic properties of H_2_O, HDO, D_2_O, and T_2_O in terms of $S\left(N,V,T,E_{\mathrm{IS}}\right)$ , *b*(*T*), and/or *B̃*_0_(*T*). An example of this is the shift in the Kauzmann temperature *T*_*K*_ among water and its isotopes which depends solely on *b*(*T*); from eq 39, Δ­(1/*T*_*K*_) = −*k*_*B*_Δ*b*(*T*_*K*_) where Δ*x* indicates the change in property *x* when going from one water isotope to another. The case of *E*_*IS*_(*T*) is particularly interesting. From eq 33 [*B̃*_1_(*T*) = 0], it follows that Δ*E*_*IS*_(*T*) = – σ^2^ Δ*b*(*T*). This means that the *E*_*IS*_(*T*) of water and its isotopes should collapse after a *T*-shift defined by Δ*β* = Δ*b*(*T*) [as long as the Gaussian approximation of the PEL holds, *T* < 280–300 K; see Figure 10]. As an example of another thermodynamic property, we consider the pressure of water and its isotopes, *P* = −(∂*F*/∂*V*)_*N*,*T*_. From eq 41

$$P\left(N,V,T\right)=-{\left(\frac{\partial E_{0}\left(N,V\right)}{\partial V}\right)}_{N}+\sigma^{2}\left(N,V\right){\left(\frac{\partial b\left(N,V,T\right)}{\partial V}\right)}_{N,T}+\beta {\left(\frac{\partial \sigma^{2}\left(N,V\right)}{\partial V}\right)}_{N}-k_{B}T{\left(\frac{\partial S\left(N,V,T,E_{\mathrm{IS}}\right)}{\partial V}\right)}_{N,T}-k_{B}T{\left(\frac{\partial {\mathrm{B̃}}_{0}\left(N,V,T\right)}{\partial V}\right)}_{N,T}-T{\left(\frac{\partial S_{\mathrm{IS}}\left(N,V,T\right)}{\partial V}\right)}_{N,T}$$ 42

Hence, Δ*P* can be obtained solely from the partial derivatives (∂/∂*V*)_*N*, *T*_ of Δ*b*, ΔS , and Δ*B̃*_0_(*N*, *V*, *T*). It would be important in the future to extend the PEL formalism to calculate the EOS of water and its isotopes (eq 42). By identifying the PEL parameters of water and its isotopes that control the location of the LLCP in H_2_O and D_2_O it would also be possible to relate the topography of the PEL with other approaches, such as the corresponding states analyses of refs 30,31 . For example, it is possible that the scaling parameters defined in refs 30,31 are related to the different PEL basins curvature of H_2_O and D_2_O.

**Figure 10:**
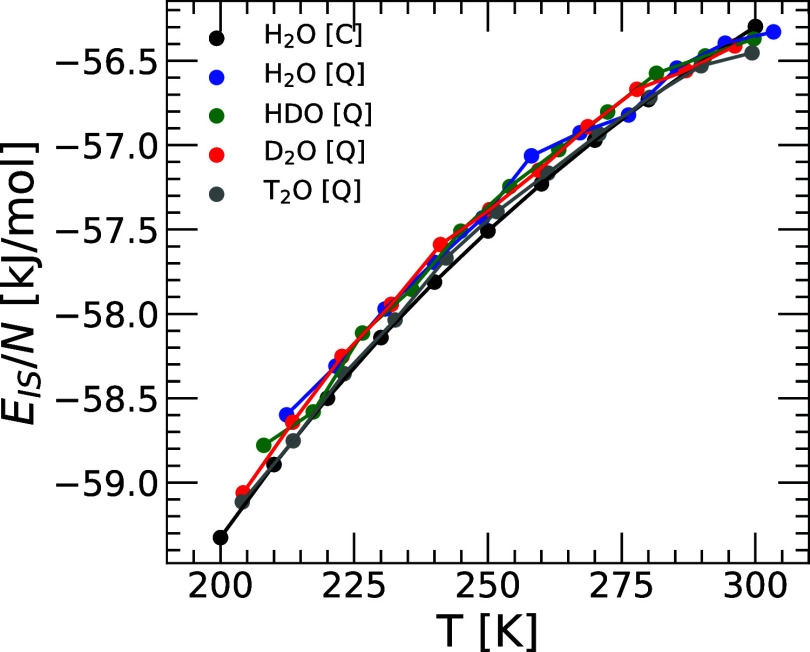
IS energy of water and its isotopes [circles, from Figure 1(b)] as a function of *T*. The *E*_*IS*_(*T*) data for the water isotopes are shifted in temperature according to Δ*β* = Δ*b* where Δ indicates the difference relative to *classical* H_2_O (Δ*x* = *x*_*isotope*_^[*Q*]^ – *x*_*H*_2_ *O*_^[*C*]^) [see text].

The PEL formalism can be useful in understanding the role of H/D concentration in H_2_O/HDO/D_2_O mixtures. The composition of these mixtures varies with the mole fraction of D present in the sample, as well as the working conditions and hence, thermodynamic properties should vary as well. For example, recent experimental work has investigated the viscosity of mixtures of H_2_O and D_2_O (and, by default, HDO),^78^ showing a systematic composition-dependent changes with increasing mole fraction of D. In this context, our results suggest that the thermodynamic properties of H_2_O/HDO/D_2_O mixtures should interpolate between the limiting cases of pure H_2_O and D_2_O. Specifically, the Helmholtz free energy of the system is given by *F* = *F*_*IS*_ + *F*_*vib*_. Since *F*_*IS*_ = *E*_*IS*_ – *TS*_*IS*_ depends only on the IS of the system, and all the H_2_O/HDO/D_2_O mixtures should have the same IS, it follows that any change due to the mole fraction of H/D is due to changes in *F*_*vib*_. It follows that, again, the differences in the H_2_O/HDO/D_2_O mixtures should arise solely from the changes in the basins shape of the RP-PEL associated with water as the mole fraction of D varies. Our results suggest that all relevant properties associated with *F*_*vib*_, such as the PEL variable S , evolve monotonically along the sequence H_2_O → HDO → D_2_O [Figures 2, 3b, 4–6], suggesting that all thermodynamic properties should evolve monotonically, from their values in H_2_O to those in D_2_O, as the mole D fraction increases. In the future, it would be useful to explore how the PEL formalism can be extended to describe such mixtures.

## Supplementary Material



## Figures and Tables

**Figure 11:**
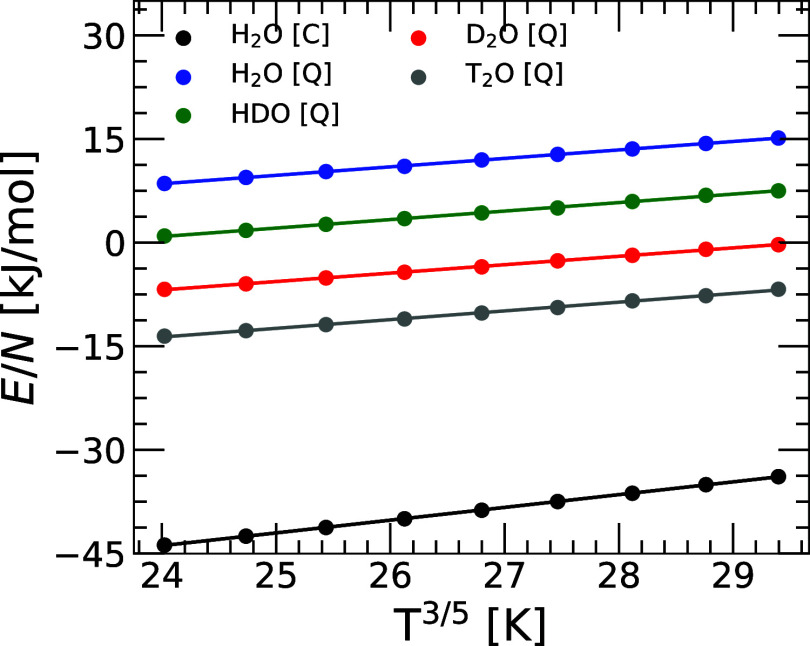
Total energy *E*(*T*) as a function of *T* ^3/5^ for q-TIP4P/F water and its isotopes at *v* = 18.0 cm^3^/mol. The circles are values of *E*(*T*) obtained from PIMD simulations and the lines are the linear fits using eq 44. For water and its isotopes, *E*(*T*) ∝*T* ^3/5^. Black circles and line are the results obtained from classical MD simulations in ref 39.

**Figure 12:**
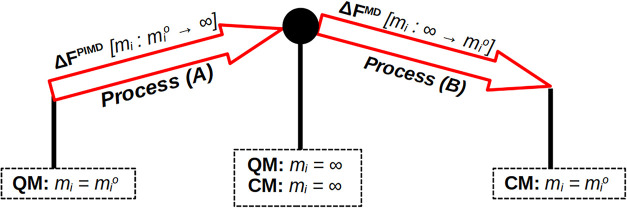
Schematic diagram showing the two-step path followed to calculate the Helmholtz free energy difference, ΔF_Q→C_, between classical and quantum H_2_O [eq 58] at constant *T*_0_ and *v.* Δ*F* ^*PIMD*^(*m*_*i*_:*m*_*i*_^0^ → ∞) is the free energy change of the quantum liquid (PIMD simulations) as the atoms masses *m*_*i*_ increase from their original mass *m*_*i*_^0^ to ∞. Similarly, Δ*F* ^*MD*^(*m*_*i*_: ∞ → *m*_*i*_^0^) is the free energy change of the classical liquid (MD simulations) as the atoms masses *m*_*i*_ decrease from ∞ to *m*_*i*_^0^.

**Figure 13:**
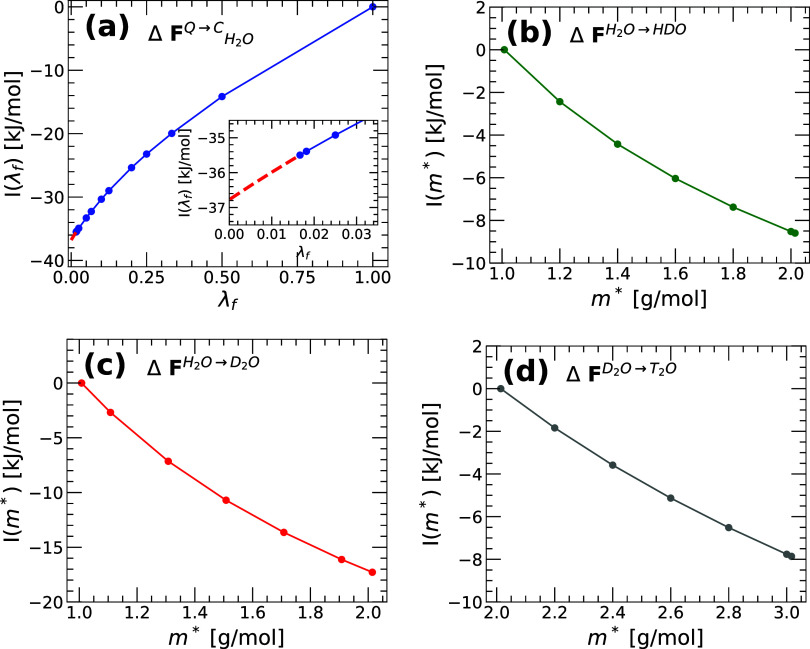
Thermodynamic integration for (a) H_2_O, (b) HDO, (c) D_2_O, and (d) T_2_O from PIMD simulations using the q-TIP4P/F water model at *T*_0_ = 260 K and *v* = 18.0 cm^3^/mol. In (a) the extrapolation of *I*(λ_*f*_) → 0 was obtained by using a straight line (red dashed line).

## Data Availability

The authors confirm that the data supporting the findings of this study are available within the article and its appendix.

## Appendix A Calculating the Entropy of q-TIP4P/F Water and its Isotopes

In this section, we describe the method used to calculate the entropy *S*(*T*) of q-TIP4P/F water and its isotopes along the isochore *v* = 18.0 g/cm^3^ using PIMD simulations. Briefly, we perform thermodynamic integration starting from the reference temperature *T*_0_ = 260 K, and use the thermodynamic relation *dS* = *dE*/*T* (at constant *v*). If the entropy of the system at the reference temperature *T*_0_ is *S*_0_ then the entropy of the system at an arbitrary temperature is given by

43
$$S\left(T\right)=S_{0}+\int_{T_{0}}^{T}\frac{1}{T^{\prime }}{\left(\frac{\partial E}{\partial T^{\prime }}\right)}_{N,V}dT^{\prime }$$

where *E* = *E*(*T*) is the total energy of the system at temperature *T* obtained from the PIMD simulations. The details for the calculation of *S*_0_ are explained below. In order to evaluate eq 43, we fit *E*(*T*) to an analytical expression. Previous computational studies for rigid and flexible water models^39,67^ have found that the potential energy, *E*_*pot*_(*T*) can be accurately described using the Tarazona approximation.^80^ In this work, we use the Tarazona approximation to fit *E*(*T*) instead of *E*_*pot*_(*T*),

44
$$E\left(T\right)=a+bT^{3/5}$$

where *a* and *b* are fitting parameters. As shown in Figure 11, for q-TIP4P/F water and its isotopes, the *E*(*T*) obtained from PIMD simulations can be fitted remarkably well using eq 44 [we also tested our results by fitting the values of *E*(*T*) from the PIMD simulations using a quartic polynomial, *E*(*T*) = ∑_*i* = 1_^4^ *c*_*i*_ *T* ^*i*^, instead of eq 44, and obtain similar results for *S*(*T*)]. Summarizing, *S*(*T*) is calculated up to the reference value *S*_0_ by first fitting *E*(*T*) using eq 44 and then integrating eq 43 to temperature *T*. The reference entropy is given by $S_{0}=\frac{E_{0}-F_{0}}{T_{0}}$ where *E*_0_ and *F*_0_ are the total energy and Helmholtz free energy at *T*_0_. While *E*_0_ is readily accessible from PIMD simulations, the calculation of *F*_0_ requires some discussion (see below).

### A.1 Helmholtz Free Energy of H_2_O at the Reference Temperature T_0_

The Helmholtz free energy *F*_0_ ≡ *F*_*Q*_(*T*_0_) of quantum H_2_O at the reference temperature *T*_0_ is calculated using thermodynamic integration over the molecular mass, following the procedure used in ref 55. The classical Helmholtz free energy at the given state point (*T*_0_ = 260 K, *v* = 18.0 cm^3^/mol), *F*_*C*_(*T*_0_), is used as the reference. Briefly, at the reference temperature *T*_0_ and given volume *v*

$$F_{Q}\left(T_{0}\right)=F_{C}\left(T_{0}\right)-\Delta F_{Q\to C}$$
45

The free energy *F*_*C*_(*T*_0_) was calculated in ref 39 using classical MD simulations of q-TIP4P/F water, and is taken from that work [*F*_*C*_(*T*_0_) for H_2_O at *T*_0_ = 260 K and *v* = 18.0 cm^3^/mol is −37.68 kJ/mol]. ΔF_Q→C_ is the free energy change when removing NQE (i.e., to convert quantum water to classical water).

Following ref 55, ΔF_Q→C_ is calculated along the two-steps defined schematically in Figure 12. The first step corresponds to process (A) where the atomic masses *m*_*i*_ (*i* = 1,2,..., 3*N*) of *quantum* water increase from their true values *m*_*i*_^0^ to ∞ [while keeping the temperature *T*_0_ and volume *v* constant]. This process involves a series of PIMD simulations where the mass of the water atoms are re-scaled (*m*_*i*_: *m*_*i*_^0^ → ∞) such that the ring-polymers collapse (*R*_*g*_ ≈ 0) for very large *m*_*i*_. The final system of process (A) is, effectively, classical water with all masses *m*_*i*_ → ∞.

The second step in Figure 12 corresponds to process (B) where the atomic masses *m*_*i*_ of *classical* water decreases from ∞ to their original values *m*_*i*_^0^ [while keeping the temperature *T*_0_ and volume *v* constant]. Process (B) involves a series of classical MD simulations where the atomic masses of the classical system with *m*_*i*_ → ∞ are re-scaled back to their original values *m*_*i*_^0^.

If Δ*F* ^*PIMD*^(*m*_*i*_: *m*_*i*_^0^ → ∞) and Δ*F* ^*MD*^(*m*_*i*_: ∞ → *m*_*i*_^0^) denote the free energy changes associated with processes (A) and (B) then [Figure 12]­

$$\Delta F_{Q\to C}=\Delta F^{\mathrm{PIMD}}\left(m_{i}:m_{i}^{0}\to \infty \right)+\Delta F^{\mathrm{MD}}\left(m_{i}:\infty \to m_{i}^{0}\right)$$
46

Equivalently

$$\Delta F_{Q\to C}=\Delta F^{\mathrm{PIMD}}\left(m_{i}:m_{i}^{0}\to \infty \right)-\Delta F^{\mathrm{MD}}\left(m_{i}:m_{i}^{0}\to \infty \right)$$
47

*Process (A)*. To calculate Δ*F* ^*PIMD*^(*m*_*i*_: *m*_*i*_^0^ → ∞), we introduce the parameter *λ* so *m*_*i*_ = *m*_*i*_^0^/*λ*. Hence, 1 ≤ *λ* ≤ 0 for *m*_*i*_^0^ ≤ *m*_*i*_ ≤ ∞. If *F* ^*PIMD*^(*λ*) is the Helmholtz free energy of the quantum water/ring polymer system [*F* ^*PIMD*^ = −*k*_*B*_ *T *ln *Q* and eq 3] when the atoms masses are replaced by *m*_*i*_ → *m*_*i*_^0^/*λ* then it can be show that

48
$$\Delta F^{\mathrm{PIMD}}\left(m_{i}:m_{i}^{0}\to \infty \right)=F^{\mathrm{PIMD}}\left(\lambda =0\right)-F^{\mathrm{PIMD}}\left(\lambda =1\right)=\int_{1}^{0}{\left(\frac{\partial F^{\mathrm{PIMD}}}{\partial \lambda }\right)}_{N,V,T}d\lambda$$

To solve this integral, we note that

49
$${\left(\frac{\partial F^{\mathrm{PIMD}}}{\partial \lambda }\right)}_{N,V,T}=-\frac{k_{B}T}{Q}{\left(\frac{\partial Q}{\partial \lambda }\right)}_{N,V,T}$$

Hence, using eq 3 (quantum water), one obtains

50
$${\left(\frac{\partial F^{\mathrm{PIMD}}}{\partial \lambda }\right)}_{N,V,T}=\left\langle \frac{dH_{\mathrm{RP}}\left(\lambda \right)}{d\lambda }\right\rangle$$

where $H_{\mathrm{RP}}\left(\lambda \right)$ is given by eq 4 and <···> indicates an ensemble average at (*N*, *v*, *T*_0_, *λ*). It follows from eqs 4 and 50 that

51
$${\left(\frac{\partial F^{\mathrm{PIMD}}}{\partial \lambda }\right)}_{N,V,T}=\frac{1}{\lambda }\left\langle KE_{Q}\left(\lambda \right)\right\rangle$$

where *KE*_*Q*_(*λ*) is the kinetic energy estimator of quantum water,^40^ i.e.,

52
$$KE_{Q}\left(\lambda \right)=\sum_{i=1}^{n}\sum_{k=1}^{n_{b}}\frac{{\left(p_{i}^{k}\right)}^{2}}{2m_{i}^{\prime }\left(\lambda \right)}-\sum_{i=1}^{n}\sum_{k=1}^{n_{b}}\frac{1}{2}k_{i}^{\mathrm{sp}}\left(\lambda \right){\left(r_{i}^{k+1}-r_{i}^{k}\right)}^{2}$$

where *m*_*i*_^′^ = *n*_*b*_ *m*(*λ*) and *k*_*i*_^*sp*^(*λ*) = *m*(*λ*)*n*_*b*_/(*β*ℏ)^2^. Therefore, from eq 51, one obtains

53
$$\Delta F^{\mathrm{PIMD}}\left(m_{i}:m_{i}^{0}\to \infty \right)=\int_{1}^{0}\frac{1}{\lambda }\left\langle KE_{Q}\left(\lambda \right)\right\rangle d\lambda$$

*Process (B)*. A similar analysis shows that for the classical system along process (B) [Figure 12]­

54
$$\Delta F^{\mathrm{MD}}\left(m_{i}:m_{i}^{0}\to \infty \right)=F^{\mathrm{MD}}\left(\lambda =0\right)-F^{\mathrm{MD}}\left(\lambda =1\right)=\int_{1}^{0}{\left(\frac{\partial F^{\mathrm{MD}}}{\partial \lambda }\right)}_{N,V,T}d\lambda$$

where

55
$${\left(\frac{\partial F^{\mathrm{MD}}}{\partial \lambda }\right)}_{N,V,T}=\left\langle \frac{dH_{C}}{d\lambda }\right\rangle$$

where $H_{C}$ is the Hamiltonian of classical water. Accordingly

56
$${\left(\frac{\partial F^{\mathrm{MD}}}{\partial \lambda }\right)}_{N,V,T}=\frac{1}{\lambda }\left\langle KE_{C}\left(\lambda \right)\right\rangle$$

where *KE*_*C*_(*λ*) is the kinetic energy estimator of classical water, ${\mathrm{KE}}_{C}\left(\lambda \right)=\sum_{i=1}^{n}\frac{{\left(p_{i}^{k}\right)}^{2}}{2m_{i}\left(\lambda \right)}$ . Therefore, from eq 54, one gets

57
$$\Delta F^{\mathrm{MD}}\left(m_{i}:m_{i}^{0}\to \infty \right)=\int_{1}^{0}\frac{1}{\lambda }\left\langle KE_{C}\left(\lambda \right)\right\rangle d\lambda =\int_{1}^{0}\frac{\left(9Nk_{B}T/2\right)}{\lambda }d\lambda$$

Combining both processes (A) and (B), eqs 53 and 57, the quantum correction is

$$\Delta F_{Q\to C}=\int_{1}^{0}\frac{1}{\lambda }{\left[\left\langle KE_{Q}\right\rangle -\frac{9N}{2}k_{B}T\right]}_{N,V,T}d\lambda$$
58

The upper integration limit in eq 58, *λ* = 0, corresponds to the infinite-mass limit which is not computationally accessible. Accordingly, we perform 2 ns-long PIMD simulations for various values of *λ* and evaluate the integral

59
$$I\left(\lambda_{f}\right)=\int_{1}^{\lambda_{f}}\frac{1}{\lambda }{\left[\left\langle KE_{Q}\right\rangle -\frac{9N}{2}k_{B}T\right]}_{N,V,T}d\lambda$$

It follows that

$$\Delta F_{Q\to C}=\underset{\lambda_{f}\;\to 0}{\lim}I\left(\lambda_{f}\right)$$
60

Figure 13(a) shows *I*(*λ*_*f*_) for various values of *λ*_*f*_ down to 0.018, which corresponds to a final mass *m*_*i*_ ≈ 1000 g/mol. To evaluate of Δ*F*_Q→C_, we extrapolate the values of *I*(*λ*_*f*_) to *λ*_*f*_ = 0 using a straight line [red dashed line in Figure 13(a)]. The resulting value of Δ*F*_*Q* → *C*_ for H_2_O, along with the corresponding values of *S*_0_ and *E*_0_ at *T*_0_ = 260 K (*v* = 18.0 cm^3^/mol) are given in Table 5.

**Table 5:** Helmholtz Free Energy *F*_0_, Total Energy *E*_0_, and the Entropy *S*_0_ for the Isotopes of q-TIP4P/F Water at *T*_0_ = 260 K and *v* = 18.0 cm^3^/mol Obtained from Thermodynamic Integration

isotope	[kJ/mol]	*F*_0_ [kJ/mol]	*E*_0_ [kJ/mol]	*S*_0_ [J/mol K]
H_2_O [Q]	Δ*F*_*H*_2_ *O* [Q] → *H*_2_ *O*[C]_ = –36.82	–0.86	13.54	55.40
HDO [Q]	Δ*F*_*H*_2_ *O* [*Q*]→*HDO*[*Q*]_ = −8.60	–9.46	5.86	58.92
D_2_O [Q]	Δ*F*_*H*_2_ *O* [*Q*] → *D*_2_ *O*[*Q*]_ = −17.28	–18.15	–1.69	63.30
T_2_O [Q]	Δ*F*_*D*_2_ *O* [*Q*] → *T*_2_ *O*[*Q*]_ = −7.86	–26.01	–8.33	68.00
H_2_O [C]		–37.68	–36.28	5.41

#### A.2 Helmholtz Free Energy of HDO/D_2_O/T_2_O at the reference temperature T_0_

The Helmholtz free energy difference between (quantum) H_2_O and its isotopes, HDO/D_2_O/T_2_O, at the reference state (*T*_0_ = 260 K, *v* = 18.0 cm^3^/mol*)* are evaluated by following a similar procedure as in process (A), point (A1) above. As an example, we consider the free energy difference between D_2_O and H_2_O. The Helmholtz free energy of D_2_O is given by

61
$$F_{D_{2}O}=F_{H_{2}O}+\Delta F_{H\to D}$$

where *F*_*H*_2_O_ is the Helmholtz free energy of H_2_O calculated in point (A1) above (including NQE) at the given (*T*_0_, *v*).

To calculate Δ*F*_*H* → *D*_ we increase the mass of the H atoms, *m*_*i*_^*H*^ (*i* = 1,2,..., 2*N*), from the corresponding true value, *m* ^*H*^ = 1.008 g/mol, to the target isotope mass *m* ^*D*^ = 2.016 g/mol. If *F*(*m*) is the Helmholtz free energy of the system when the H masses are *m*_*i*_^*H*^ = *m* then, as discussed above for process (A) in point (A1)

$$\Delta F_{H\to D}=F(m^{D})-F\left(m^{H}\right)=\int_{m^{H}}^{m^{D}}{\left(\frac{\partial F\left(m\right)}{\partial m}\right)}_{N,V,T}\mathrm{dm}$$
62

Since *F* = −*k*_*b*_ *T*  ln *Q* where *Q* is the canonical partition function of the ring-polymer system, eq 3, one can show that

63
$${\left(\frac{\partial F\left(m\right)}{\partial m}\right)}_{N,V,T}=\left\langle \frac{dH_{\mathrm{RP}}\left(m\right)}{\mathrm{dm}}\right\rangle =-\frac{\left\langle KE^{H}\left(m\right)\right\rangle }{m}$$

where *KE* ^*H*^(*m*) is the estimator for the kinetic energy of the H atoms

64
$$KE^{H}\left(m\right)=\sum_{i=1}^{2N}{\;}^{\prime }\sum_{k=1}^{n_{b}}\frac{{\left(p_{i}^{k}\right)}^{2}}{2m^{\prime }\left(m\right)}-\sum_{i=1}^{2N}\sum_{k=1}^{n_{b}}\frac{1}{2}k_{H}^{\mathrm{sp}}\left(m\right){\left(r_{i}^{k+1}-r_{i}^{k}\right)}^{2}$$

where ∑_*i*_^′^ indicates sum over all the 2*N* hydrogen atoms in the system, *m*′ = *n*_*b*_ *m*, and *k*_*H*_^*sp*^(*m*) = *m n*_*b*_/(βℏ)^2^.

The integral in eq 62 is evaluated by performing 2 ns-long PIMD simulations for six different H atom masses *m* linearly spaced so *m* ^*H*^ ≤ *m* ≤ *m* ^*D*^. Figure 13(c) shows *I*(*m**) defined as

65
$$I\left(m^{*}\right)=\int_{m^{H}}^{m^{*}}{\left(\frac{\partial F\left(m\right)}{\partial m}\right)}_{N,V,T}\mathrm{dm}$$

From eq 62, Δ*F*_*H* → *D*_ = *I*(*m** = *m* ^*D*^). The calculated values of *F*_0_ ≡ *F*_*D*_2_O_, total energy *E*_0_, and entropy *S*_0_ for D_2_O are reported in Table 5.

A similar procedure is applied to calculate the Helmholtz free energy of T_2_O. In this case, however, we start from the reference free energy of D_2_O, instead of H_2_O. The results for T_2_O are presented in Figure 13(d) and Table 5.

For the case of HDO, we repeat the same procedure above to evaluate *F*_*D*_2_O_ starting with H_2_O as a reference system. However, in this case, we only alter the mass of one of the H atoms of the water molecules (e.g, in eq 64, the ∑_*i*_^′^ is limited to only *N* hydrogen atoms). The results for HDO are presented in Figure 13(b) and Table 5.

## Notes

Calculation of *S*_*IS*_(*T*) for quantum H_2_O using eq 13; comparison of results with PEL parameters {α, *E*_0_, σ^2^} constrained to their classical values and unconstrained, as in previous classical PEL studies (PDF)
