# Supplementary material for: Understanding Isotope Substitution Effects in Water Using the Potential Energy Landscape Formalism for Quantum Liquids
Source: J Chem Theory Comput. 2025 Nov 18;21(23):11931–50. doi: 10.1021/acs.jctc.5c01325 (PMC12874375; doi:10.1021/acs.jctc.5c01325)
Supplement: Supplementary file 1 [file ct5c01325_si_001.pdf]

# Supplementary Material for 'Understanding Isotope Substitution Effects in Water using the Potential Energy Landscape Formalism for Quantum Liquids'

Ali Eltareb[1,2,*], Yang Zhou[1,2,*], Gustavo E. Lopez[3,4,*], and Nicolas Giovambattista[1,2,4*]

[1] *Department of Physics, Brooklyn College of the City University of New York, Brooklyn, NY 11210, United States*

[2] *Ph.D. Program in Physics, The Graduate Center of the City University of New York, New York, NY 10016, United States*

[3] *Department of Chemistry, Lehman College of the City University of New York, Bronx, NY 10468, United States*

[4] *Ph.D. Program in Chemistry, The Graduate Center of the City University of New York, New York, NY 10016, United States*

[*] aeltareb@gradcenter.cuny.edu, yzhou4@gradcenter.cuny.edu, gustavo.lopez1@lehman.cuny.edu, ngiovambattista@brooklyn.cuny.edu

## I. CONFIGURATIONAL ENTROPY AND THE NATURE OF THE PEL PARAMETERS $\{\alpha, E_0, \sigma^2\}$

The PEL parameters $\{\alpha, E_0, \sigma^2\}$ define the distribution of IS in the PEL of water, $\Omega_{IS}(e_{IS})$, and the configurational entropy, $S_{IS}(T)$ [Eqs. 12 and 13 of the main manuscript]. In the main manuscript, we argued that $\Omega_{IS}(e_{IS})$, $S_{IS}(T)$, and hence, the PEL parameters $\{\alpha, E_0, \sigma^2\}$ of classical (MD simulations) and quantum water (PIMD simulations) must be identical to one another. Here, we compare the resulting $S_{IS}(T)$, reported in the main manuscript, with the $S_{IS}(T)$ that results when the PEL parameters $\{\alpha, E_0, \sigma^2\}$ are left unconstrained and are calculated using the standard method employed previously in PEL studies based on classical MD simulations (e.g, Refs. [1–6]).

In previous PEL studies [1–6], the PEL parameters $E_0$ and $\sigma^2$ are evaluated by fitting the values of $E_{IS}(T)$ obtained from the computer simulations using Eq. 18 of the main manuscript. In addition, anharmonic corrections are modeled using Eq. 28 with $\tilde{B}_1 = 0$, and Eq. 37 with $c_{0,0} = 0$. This allows one to evaluate the parameter $\alpha$ by fitting the numerical values of $S_{IS}(T) = S(T) - S_{vib}^{harm}(T) - S_{vib}^{anh}(T)$ using Eq. 13 of the main manuscript. The values of $S_{IS}(T)$ evaluated in this way for the case of quantum q-TIP4P/F water (PIMD simulations) are shown in Fig. S1 (blue symbols: PIMD simulations; blue line: Eq. 13 of the main manuscript). Also included in Fig. S1 are the values of $S_{IS}(T)$ for quantum (red symbols/lines) and classical (black symbols/lines) $H_2O$ taken from Fig. 7(d) of the main manuscript.

Fig. S1 indicates that following the method of Refs. [1–6], i.e., allowing $\{\alpha, E_0, \sigma^2\}$ to be unconstrained (blue symbols/line), leads to unphysical results. Indeed, from a physics perspective, the $S_{IS}(T)$ of classical and quantum water must converge at high temperatures, as NQE become negligible. However, this is not the case when using the method of Refs. [1–6] (blue and black lines/symbols). Requiring that the PEL parameters $\{\alpha, E_0, \sigma^2\}$ are identical in quantum and classical water does not present this problem (red and black lines/symbols).

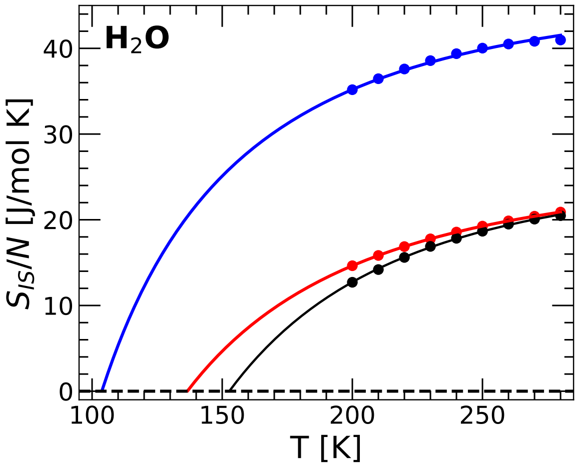


FIG. S1. Comparison of the configurational entropy, $S_{IS}(T)$, as a function of temperature at $v$ = 18.0 cm$^3$/mol for $H_2O$ obtained from classical MD and path-integral computer simulations (circles). The red line corresponds to the values of $S_{IS}(T)$ for quantum $H_2O$ reported in Fig. 7(d) of the main manuscript based on Eq. 13 with the PEL parameters $\{\alpha, E_0, \sigma^2\}$ considered to be identical to the corresponding parameters of classical water (taken from Ref. [2]). The blue line is the $S_{IS}(T)$ for quantum $H_2O$ predicted by Eq. 13 where $\{\alpha, E_0, \sigma^2\}$ are treated as free parameters, calculated using the method in Refs. [2, 3]. The values of $S_{IS}(T)$ for classical $H_2O$ are included and indicated by black circles and lines (taken from Ref. [2]). The red line (but not the blue line) converges to the classical values of $S_{IS}(T)$ (black circles/line) at high temperatures, as one would expect, consistent with the PEL parameters $\{\alpha, E_0, \sigma^2\}$ being identical for classical and quantum water; see text.

## II. REFERENCES


[1] F. Sciortino, E. La Nave, and P. Tartaglia, Phys. Rev. Lett. **91**, 155701 (2003).

[2] A. Eltareb, G. E. Lopez, and N. Giovambattista, J. Chem. Phys. **160**, 154510 (2024).

[3] P. H. Handle and F. Sciortino, J. Chem. Phys. **148**, 134505 (2018).

[4] S. Sastry, Nature **409**, 164 (2001).

[5] F. Sciortino, J. Stat. Mech. **2005**, P05015 (2005).

[6] A. Scala, F. W. Starr, E. La Nave, F. Sciortino, and H. E. Stanley, Nature **406**, 166 (2000).
